# The *razABCD* regulatory island links azalomycin F biosynthesis in *Streptomyces iranensis* to orsellinic acid production in the fungus *Aspergillus nidulans*

**DOI:** 10.1016/j.jbc.2026.113572

**Published:** 2026-09-18

**Authors:** Lukas M. Zehner, Simone Edenhart, Peter Hortschansky, Maira Rosin, Sascha Schäuble, Gianni Panagiotou, Tina Netzker, Volker Schroeckh, Axel A. Brakhage

**Affiliations:** 1 Department of Molecular and Applied Microbiology, Leibniz Institute for Natural Product Research and Infection Biology (Leibniz-HKI), Jena, Germany; 2 Institute for Microbiology, Friedrich Schiller University Jena, Jena, Germany; 3 Department of Microbiome Dynamics, Leibniz Institute for Natural Product Research and Infection Biology (Leibniz-HKI), Jena, Germany

**Keywords:** Actinobacteria, *Aspergillus*, bacterial transcription, DNA binding protein, transcription factor, LuxR, natural product biosynthesis, microbial communication

## Abstract

*Streptomyces iranensis* induces the production of orsellinic acid and a range of its derivatives in the fungus *Aspergillus nidulans*. The arginoketide azalomycin F was identified as the bacterial signaling molecule that activates otherwise silent biosynthetic gene clusters (BGCs) in diverse fungi. To date, however, little is known about the regulatory mechanisms governing the azalomycin F BGC, beyond the requirement of the regulatory gene *bldA*. Here, building on the known role of *bldA*, we report the discovery of a regulatory island, *razABCD*, encoding four LuxR-family transcriptional regulators. Through the generation of deletion mutants and subsequent analysis of azalomycin F production, RazA, RazB, and RazD were identified as key regulators of the azalomycin F BGC. This regulatory island thereby governs the induction of the orsellinic acid BGC in *A. nidulans*. The DNA-binding motif of RazD (5′-CTAGGGAWTY-3′) was defined using electrophoretic mobility shift assays (EMSAs) and surface plasmon resonance analyses with the purified DNA-binding domain of RazD. Molecular genetic analyses further revealed that *razA* and *razB* act hierarchically upstream of *razD*. Collectively, our findings establish a regulatory link between the control of a bacterial biosynthetic gene cluster and the activation of a silent fungal BGC, providing mechanistic insight into interkingdom metabolic communication.

Natural products (NPs) are low molecular weight compounds that are produced by many microorganisms, including filamentous fungi (1). A number of these compounds have found application in medicine or industry, for example, as therapeutic agents, fungicides, or insecticides (2). Examples of this include the antibiotics cephalosporin C and penicillin G, the immunosuppressant cyclosporin A, and the antifungal agent griseofulvin (3). The biosynthesis genes for these compounds are often physically linked in biosynthetic gene clusters (BGCs) (4). Analyses of sequenced fungal genomes using search programs such as SMURF or AntiSMASH allow the identification of BGCs (5, 6). Based on such analyses, the model filamentous fungus *Aspergillus nidulans* contains at least 66 putative BGCs. However, most of these have not yet been assigned to NPs (7), as many BGCs remain inactive or are expressed at low levels under standard laboratory conditions (3). These BGCs are only active when the respective environmental stimulus is present, such as a microbial partner (8, 9, 10).

One of the earliest examples of this is the activation of the cryptic orsellinic acid (*ors)* BGC in *A. nidulans* when the fungus is cultured alongside the soil-dwelling bacterium *Streptomyces rapamycinicus* or a closely related species, *Streptomyces iranensis* (11, 12). Recently, we identified a group of polyketides called arginoketides that induce the silent fungal *ors* BGC in response to a bacterial signal (11). The inducing arginoketides produced by *S. iranensis* are azalomycin F derivatives (11, 13). While the azalomycin F (*azl*) BGC has been investigated in relation to the biosynthesis of azalomycin F (13, 14, 15, 16, 17), the regulation of the *azl* BGC has not yet been elucidated.

Previously, we demonstrated that the *bald* (*bld*) family of regulatory genes is essential for producing azalomycin F (11). These genes regulate key steps in the later stages of the *Streptomyces* life cycle—a developmental phase in which most NPs are produced — and thus substantially impact *Streptomyces iraniensis* NP metabolism (18, 19, 20). A notable feature of this regulatory circuit is the *bldA* gene, which encodes the tRNA^Leu^. This tRNA is unique in *S. iranensis* in its ability to efficiently translate the UUA codon into leucine (21), a regulatory feature that is also found in many other streptomycetes (22).

Here, based on the knowledge of *bldA* we discovered the novel regulatory island *razABCD*, that spans four genes, all of which encode LuxR-family transcriptional regulators. LuxR-type transcription factors have been shown to be involved in diverse regulatory processes, like quorum sensing, but also in the regulation of motility and biofilm formation, stress response and NP metabolism (23, 24). Detailed genetic and biochemical characterization of the *razABCD* island led to the identification of RazD as directly binding to the azalomycin F BGC and as being epistatic to RazA and RazB.

## Results

### Identification of the regulatory island *razABCD* regulating the azalomycin F BGC

To identify regulators of the azalomycin F biosynthesis, we started with our previous observation that an *S. iranensis* Δ*bldA* deletion mutant was not able to induce the fungal *ors* BGC (11). The gene *bldA* encodes the only tRNA in most streptomycetes capable of effectively translating the TTA codon to leucine (21, 22). Thus, it was conceivable that either genes containing a TTA codon are involved in the regulation of the azalomycin F BGC and/or the azalomycin F biosynthesis genes encode a TTA codon. Since a transcriptome analysis of the *S. iranensis* Δ*bldA* deletion mutant revealed that the transcription of the azalomycin F BGC was drastically reduced (11), this finding suggested that the expression of the encoding BGC relies on a transcription factor that contains a TTA-encoded leucine. Therefore, we screened the genome of *S. iranensis* (25) for genes that met three criteria: (i) they contain at least one TTA codon; (ii) they encode putative transcriptional regulators; and (iii) have homologs in both *S. rapamycinicus* DSM49375 and *Streptomyces violaceusniger* TU4113, which are also known producers of azalomycin F (10, 11). This way, we identified eleven genes fulfilling these criteria (Table 1). Deletion strains of these genes were generated by their substitution with an apramycin resistance cassette *via* homologous recombination. Deletions were verified by PCR, and the capability of the generated knockout mutants to produce azalomycin F was tested *via* HPLC-MS analysis.

**Table 1:** Putative *S. iranensis* Genes Involved in the Regulation of the Azalomycin F Biosynthesis

GENE LOCUS TAG	Number of TTA codons	GENE NAME	REGULATOR family
*SIRAN_RS02585 (SIRAN679)*	≥ 2		SARP-LAL
*SIRAN_RS02845 (SIRAN733)*	≥ 2		AFSR-DNRI-REDD REGULATOR
*SIRAN_RS04455 (SIRAN1070)*	≥ 2		SARP
*SIRAN_RS04785 (SIRAN1137)*	1	*RAZB*	LAL
*SIRAN_RS04790 (SIRAN1138)*	1	*RAZA*	LAL
*SIRAN_RS06950 (SIRAN1583)*	1		SARP
*SIRAN_RS15610 (SIRAN3352)*	1		HTH-ARSR
*SIRAN_RS24225 (SIRAN5116)*	1		HTH-LYSR
*SIRAN_RS36220 (SIRAN7555)*	1		PUCR (PURINE CATABOLISM)
*SIRAN_RS36840 (SIRAN7683)*	1		HTH-LYSR
*SIRAN_RS46670 (SIRAN9718)*	≥ 2		HTH-ARAC

They contain at least one TTA codon, encode a transriptional regulator and have homologs in other azalomycin F producers. The first column lists both gene locus tags assigned to each gene, according to the NCBI annotations GCF_042466515.1 and in brackets LK022848.1.

Deletion strains could be generated for nine of these genes except for the genes *SIRAN_RS15610* and *SIRAN_RS36220*, likely because their deletion is lethal. Of all nine deletion strains tested, only those for the genes *SIRAN_RS04790* and *SIRAN_RS04785*, located next to each other on the chromosome (Fig. 1), showed no azalomycin F production anymore in the culture supernatant when measured *via* HPLC-MS (Fig. 1).

**Figure 1:**
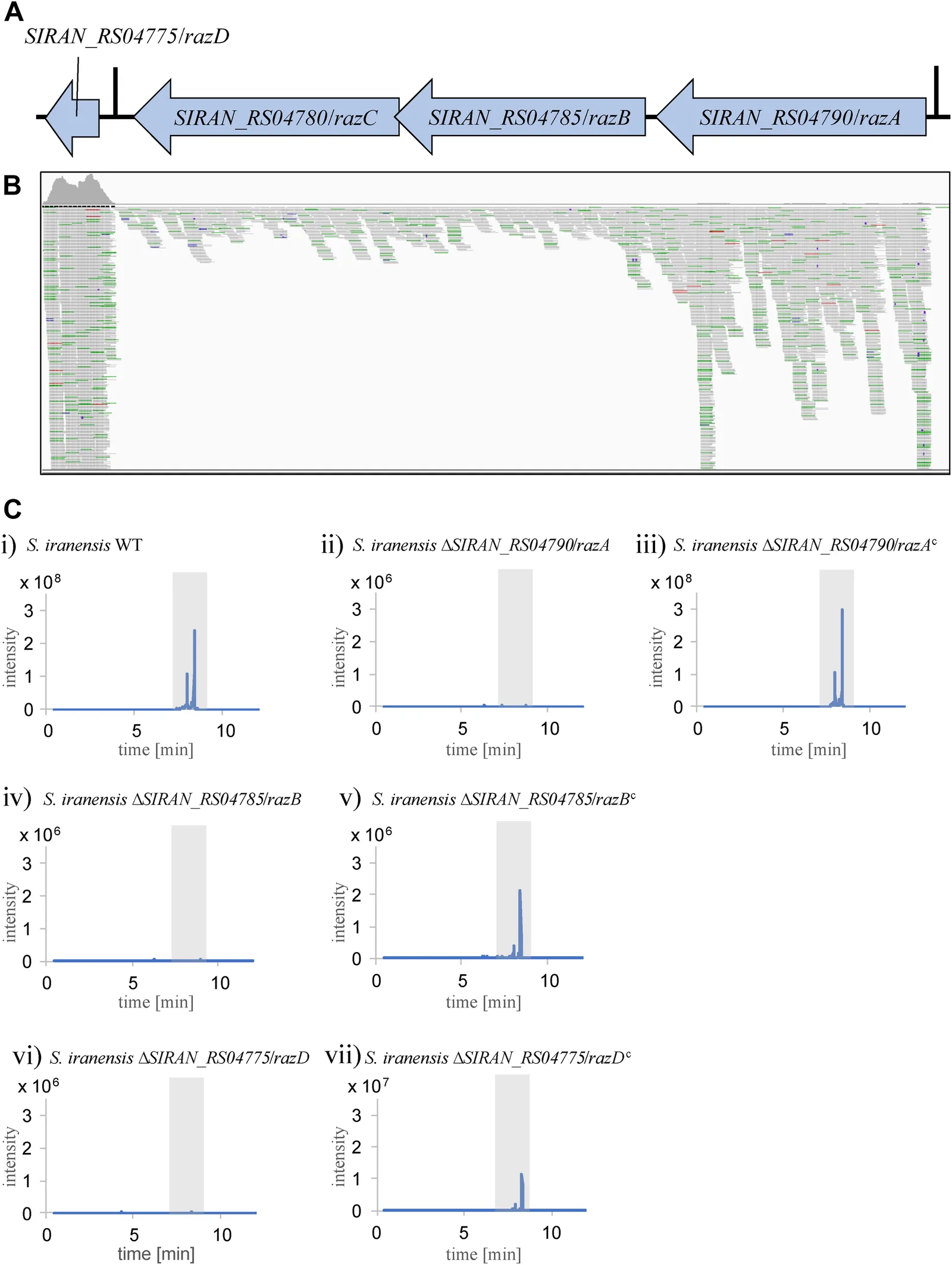
**Regulatory island harboring Raz transcription factor genes, transcriptomic reads covering these genes and azalomycin F3 production of deletion mutants.***A*, schematic depiction of the four transcription factor genes. *razA* (*SIRAN_RS04790*) and *razB* (*SIRAN_RS04785*) each contains one TTA-codon and is therefore *bldA*-dependent. The open reading frames of *razC* (*SIRAN_RS04780*) and *razB* overlap by 5 base pairs, indicating that they might be transcribed as an operon. *B*, transcriptomic reads of *S. iranensis* wild type were mapped to the genome of *S. iranensis* using the Integrative Genomic Viewer (IGV), in order to assess the levels of transcriptional activity. *razB* and *razC* showed the same level of mRNA steady-state levels, which indicated that they might be part of an operon. *razD* (*SIRAN_RS04775*) showed considerably higher transcript levels than the other three transcription factor genes and is separated by an intergenic region reflected by a gap of transcript coverage. This suggests that *razD* is transcribed separately from the other three transcription factors. *C*, extracted ion chromatograms of azalomycin F3 (*m/z* 1068 [M + H] ^+^) (marked in grey) from HPLC-MS analyses of culture supernatants from *S. iranensis* wild type (WT) and deletion mutants. (i) *S. iranensis* WT, (ii) deletion mutant Δ*SIRAN_RS04790*/*razA,* (iii) complemented *S. iranensis strain* Δ*SIRAN_RS04790*/*razA*^c^, (iv) Δ*SIRAN_RS04785/razB,* (v) complemented Δ*SIRAN_RS04785*/*razB*^c^, (vi) Δ*SIRAN_RS04775/razD,* and (vii) Δ*SIRAN_RS04775*/*razD*^c^.

We focused on genes *SIRAN_RS04785* and *SIRAN_RS04790*, which encode putative LuxR family transcriptional regulators. *SIRAN_RS04785* is translated into a protein of 918 amino acids (aa) (NCBI annotation NZ_LK022848.1) or 902 aa (annotation LK022848.1), respectively, while SIRAN_RS04790 encodes 966 aa. The genes are located adjacent to each other on the chromosome, but are separated by a short intergenic region of about 58 or 106 bp, depending on the annotation used (Fig. 1). This suggested that *SIRAN_RS04785*, which lies downstream of *SIRAN_RS04790,* is transcribed from its own promoter (Fig. 1). Further, *SIRAN_RS04785* overlaps with *SIRAN_RS04780*, which codes for another LuxR-family transcriptional regulator. Thus, these genes might form an operon. A fourth, smaller LuxR-family transcriptional regulator (*SIRAN_RS04775*), 228 aa is located downstream of *SIRAN_RS04785* and *SIRAN_RS04780* (Fig. 1). Homologous genes of the identified LuxR-type regulators were also found clustered in the genomes of other azalomycin F-producing *Streptomyces* spp. like *S. violaceusniger* TÜ 4113 and *S. rapamycinicus* SRMK07. The comparison of the annotated open reading frame (ORF) of *SIRAN_RS04775* and its respective homologs *Strvi_5061* of *S. violaceusniger* and *LJB45_00075* of *S. rapamycinicus* revealed that there are different putative start codons in *S. iranensis* and thus different protein lengths are possible. According to a previous annotation of *SIRAN_RS04775*, the SIRAN1135 ORF (GenBank CDR03258.1) of *S. iranensis* was for example, slightly shorter, with a deduced protein of 193 aa rather than 202 aa as for the other two homologs in *S. violaceusniger* and *S. rapamycinicus*. However, in *S. iranensis*, an ORF consisting of a 202-aa-long protein or even one with 220 aa, which would also contain a TTA-encoded leucine at position 5, are conceivable. Therefore, we generated an *S. iranensis SIRAN_RS04775* deletion strain. Similar to the deletions of *SIRAN_RS04785* and *SIRAN_RS04790,* the deletion of *SIRAN_RS04775* led to the loss of azalomycin F production in *S. iranensis* (Fig. 1). To verify that all three genes are involved in the regulation of the azalomycin F biosynthesis, we complemented the deletion strains by introducing intact copies of the respective genes by using the integrative plasmid pIJ10257 (26).

For all complemented deletion strains, azalomycin F production was restored to various extents (Fig. 1). Collectively, this shows that all three LuxR regulators play a major role in the regulation of azalomycin F biosynthesis. Therefore, we named these genes regulators of azalomycin F biosynthesis *razA* (*SIRAN_RS04790*), *razB* (*SIRAN_RS04785*), and *razD* (*SIRAN_RS04775*) (Fig. 1). We also included *SIRAN_RS04780* as *razC*, as it is likely part of this conserved regulatory island of LuxR regulators in azalomycin F-producing strains. However, we were not able to generate a deletion strain of *razC.* One possible explanation could be that deletion of the gene is lethal.

### RazD controls the expression of the azalomycin F BGC

To identify which of the three LuxR regulators controls the expression of the azalomycin F biosynthesis genes, we performed transcriptome analyses of Δ*razD*, Δ*razB* and Δ*razA* which revealed in all three deletion strains a strong decrease in transcript levels of all azalomycin F core biosynthetic genes compared to the wild type (Fig. 2). The reads per kilobase million (RPKM) values obtained from *S. iranensis* wild type suggested that *razD* is expressed more strongly than *razC*, *razB* or *razA* (Fig. 1*B*). Furthermore, mapping of the transcriptomic reads against the *S. iranensis* genome using the Integrative Genomic Viewer software IGV) showed (27, 28) that *razD* is separated from the three other genes of the regulatory island by a transcript gap (Fig. 1*B*). This suggests that *razD* is regulated independently of the other genes of the regulatory island. The RPKM values obtained by the transcriptome analysis indicated that *razD* was highly expressed in the *S. iranensis* wild type. However, there was barely any transcript of this gene detectable in the three deletion strains. While this is to be expected for the Δ*razD* mutant, it was interesting to notice that the deletion of *razA* and *razB* also negatively affected the mRNA steady-state level of *razD* (Fig. 3*A*). However, deletion of *razA* and *razB* had only a minor effect on each other’s expression, nor was their expression levels influenced by deletion of *razD.* We thus concluded that there must be a hierarchy amongst these transcription factors and that both RazA and RazB positively influence the transcription of *razD*. Furthermore, we assumed that neither RazA nor RazB directly regulates the expression of the azalomycin F BGC, although they are essential for its transcription in general. By contrast, RazD seemed to be at the bottom of the regulatory cascade and was therefore considered a direct regulator of the *azl* BGC. To prove this hypothesis, we conducted a cross-complementation experiment in which the deletion strains *S. iranensis* Δ*razA* and *S. iranensis* Δ*razB* were complemented with pIJ-*razD,* an integrative plasmid that contains *razD* under control of the constitutive *ermE*∗ promoter. After cross-complementation, we detected azalomycin F production in both strains, while control strains, which were only transformed with the empty vector, did not produce any azalomycin F (Fig. 3, *B*–*E*). These results implied that RazD is the main regulator of the azalomycin F biosynthesis and positively regulates the transcription of the BGC, while RazA and RazB positively regulate the transcription of *razD*.

**Figure 2:**
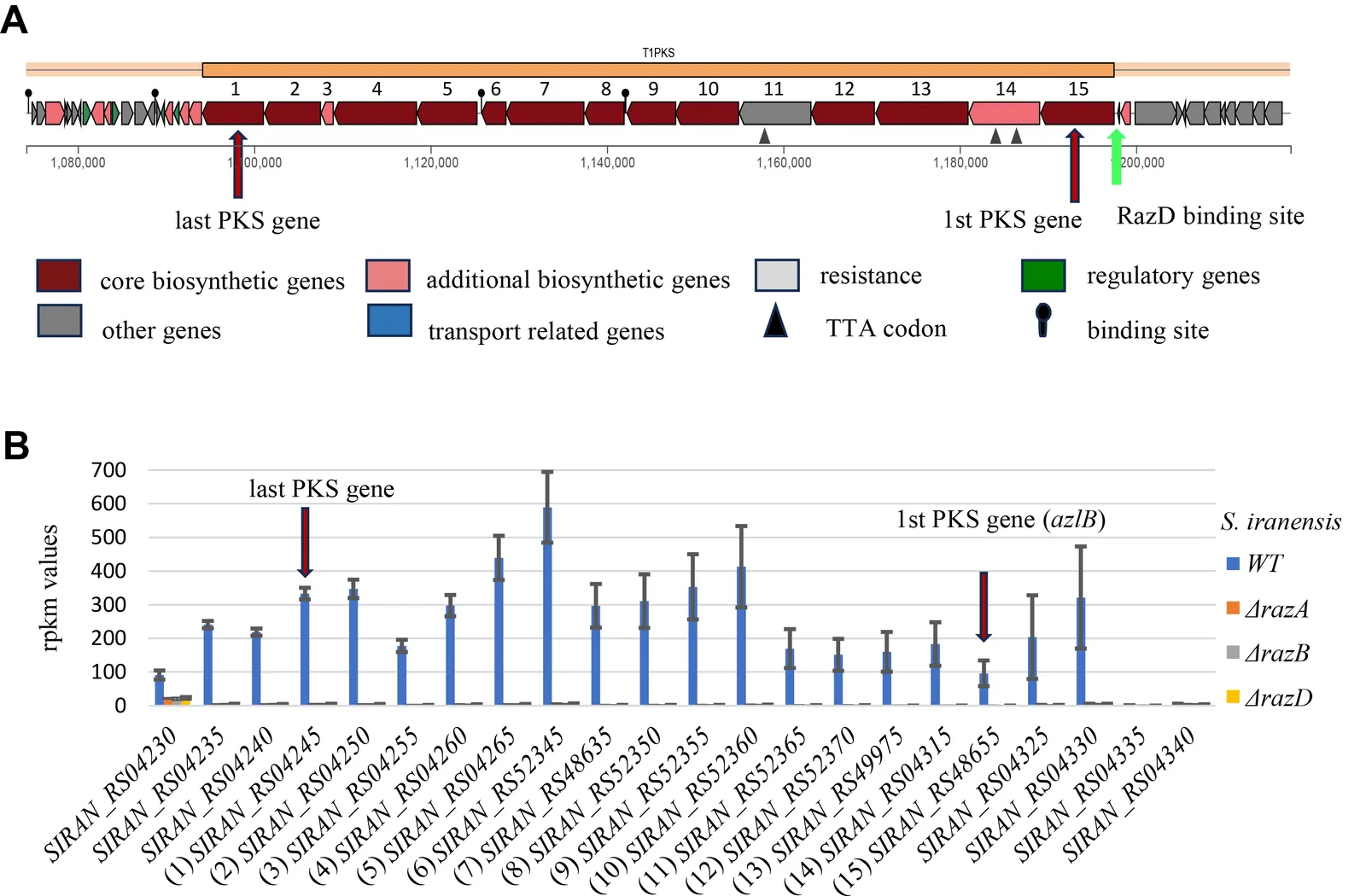
**Azalomycin F BGC overview and gene expression of the azalomycin F BGC in different *S. iranensis* strains.***A*, Azalomycin F BGC of *S. iranensis* as predicted by AntiSMASH version 8.0.4 (48) with additional arrows indicating the discovered RazD-binding site (*green*) and the first and last PKS gene of the cluster (*dark red*). The AntiSMASH algorithm has predicted putative binding sites for other known transcription factors, which are marked. Their relevance has yet to be proven. *B*, gene expression values are given as mean reads per kilobase million (RPKM) of three replicates with standard deviation of the WT and deletion mutants of *S. iranensis*. The numbers in brackets, ranging from 1 to 15, indicate the correspondence between the RPKM values and the genes in the azalomycin F gene cluster schematic.

**Figure 3:**
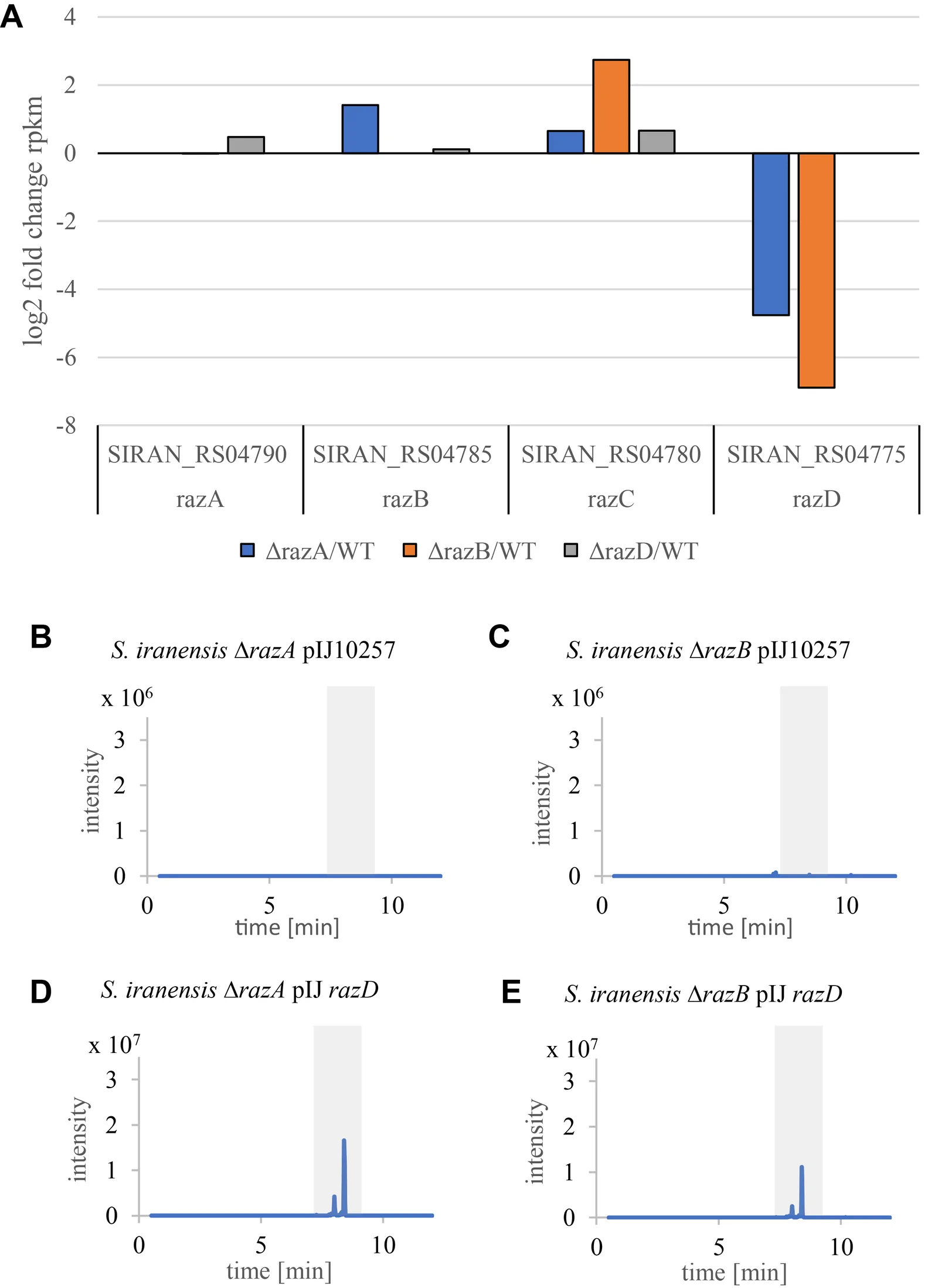
**RT-qPCR analysis and extracted ion chromatograms of azalomycin F3 (m/z 1068 [M + H] +) from an HPLC-MS analysis of culture supernatants of *S. iranensis*.***S. iranensis* deletion mutants ΔrazA and ΔrazB were cross-complemented with the plasmid pIJ *razD* that contains the LuxR regulator *razD* under the control of the constitutive promoter *ermE*∗. *A*, change in expression levels of the indicated transcription factor genes detected in the Δ*razA*, Δ*razB*, and Δ*razD* deletion mutants compared with the wild type. In contrast to the deletion strains with the empty vectors (*B* and *C*), the cross-complemented mutants (*D* and *E*) produce the azalomycin F complex (marked in *gray*).

### RazD binds to the promoter region of *azlB*

We reasoned that the LuxR-type regulator RazD directly binds a promoter region of the azalomycin F BGC. Therefore, electrophoretic mobility shift assays (EMSAs) were conducted, in which the putative promoter region of *azlB,* coding for the first polyketide synthase (PKS) gene of the azalomycin F BGC, was tested for binding by the RazD protein. However, in many LuxR-type transcription factors, the helix-turn-helix (HTH) domain is not able to bind the target DNA motif unless the respective ligand or cofactor is bound (29, 30, 31). Binding the respective cofactor leads to a conformational change of the protein, which makes the HTH domain accessible to DNA. Since the putative inducer activating RazD is unknown and cannot be added *in vitro*, we only purified a truncated version of RazD. It contained the DNA-binding HTH motif, which was N-terminally fused to a maltose-binding protein (MBP) tag. Since the low molecular mass of the RazD^HTH^ domain (10.29 kDa) was expected to result in a rather small band shift in EMSA experiments, the fusion protein retaining the MBP tag was purified and used for EMSA experiments (Fig. S1). In a first set of experiments, we tested multiple intergenic regions within the *azl* BGC for binding by the MBP-RazD^HTH^ fusion protein. For this purpose, fluorescence-labeled DNA fragments were generated by PCR, and the MBP-RazD^HTH^ protein was added in increasing amounts (up to 2 μM) to assess its binding affinities. To exclude unspecific binding of MBP-RazB^HTH^ to DNA, the unrelated gene *SIRAN_RS16895*, which showed no difference in expression when *razD* was deleted (log2-fold change Δ*razD*/WT: 0.081), was PCR-amplified with fluorescently labeled oligonucleotides as a control. No visible band shift was observed with this negative control (NC) DNA fragment in EMSAs up to a concentration of 2 μM of added protein, indicating that MBP-RazD^HTH^ does not bind DNA non-specifically in this experimental setup (Fig. 4).

**Figure 4:**
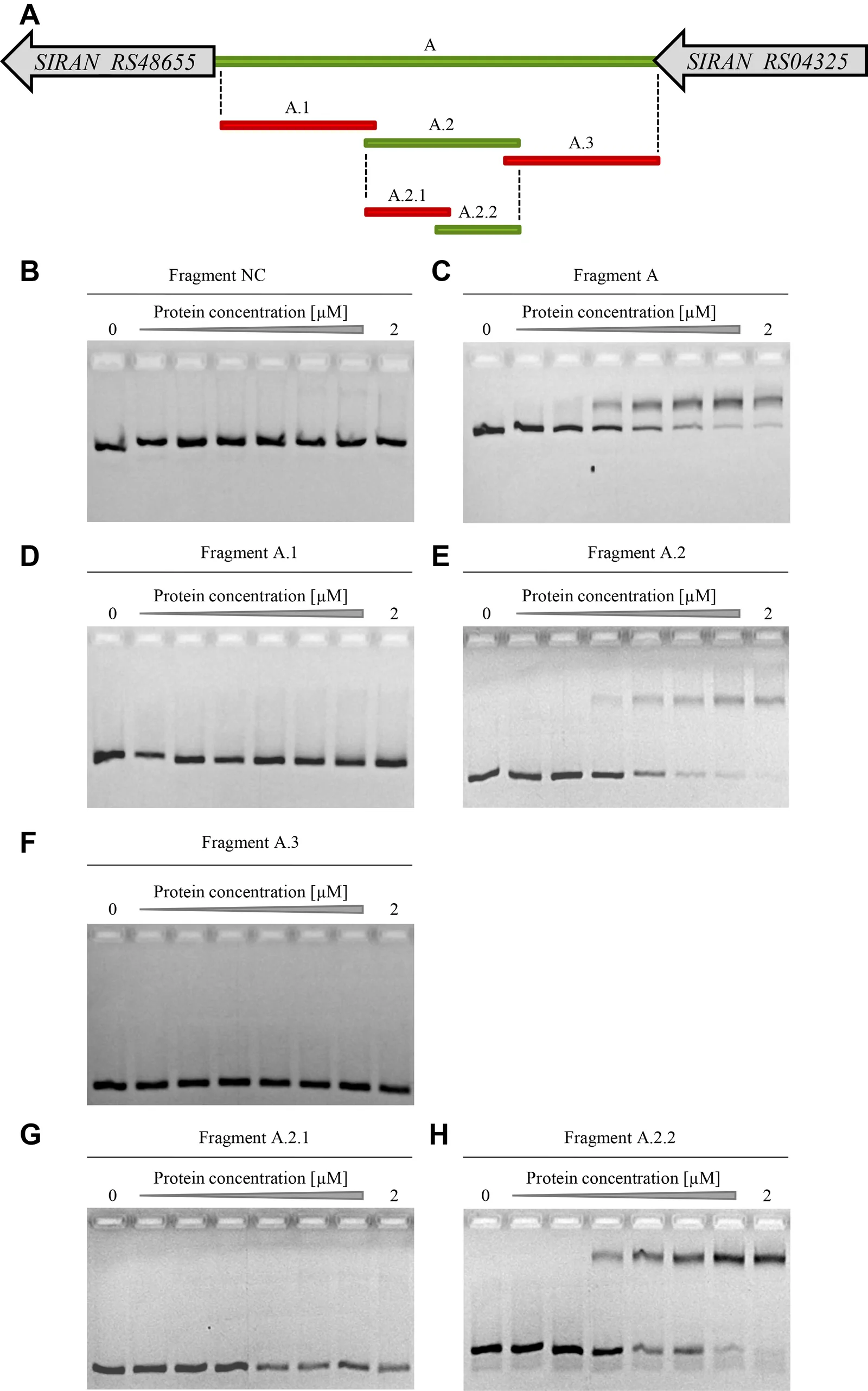
**Schematic of DNA fragments in the putative promoter region of the azalomycin F gene cluster used for electrophoretic mobility shift assays (EMSAs) and the respective EMSAs.** An MBP-RazD^HTH^ fusion protein was purified and incubated with fluorescently labelled DNA probes of a putative promoter region. *A*, schematic depiction of the tested DNA probes and their location in the intergenic region upstream of the azalomycin F gene cluster. DNA fragments that showed binding by the MBP-RazD^HTH^ fusion protein are depicted in *green*, while fragments that did not show binding are depicted in red. *B*) As negative control (NC) the unrelated gene *SIRAN_RS16895* was used. *C*, fragment A, the upstream region of *SIRAN_RS48655*, the first PKS of the azalomycin F gene cluster, showed binding. This fragment A was split into smaller fragments (*D*) A.1, (*E*) A.2, and (*F*) A.3, of which only A.2 showed binding. Fragment A2 then again was subdivided into (*G*) A.2.1, which did not show binding and (*H*) fragment A.2.2, which showed binding to MBP-RazD^HTH^.

Next, putative promoter regions within the azalomycin F BGC were tested for binding of the MBP-RazD^HTH^ fusion protein (Fig. 4). However, band shifts were only observed for one of the tested DNA probes, the 420 bp DNA fragment A amplified from the intergenic region upstream of *SIRAN_RS48655 (azlB),* the first PKS of the *azl* BGC (Fig. 4*C*). Band shifts were detected at protein concentrations of 0.6 μM and higher. The affinity of a protein-DNA interaction is commonly described by the dissociation constant (K_D_), which is the protein concentration at which half of the available DNA is bound by the respective protein. The K_D_ value can be approximated from the band shift patterns observed in EMSAs by estimating the protein concentration at which the free DNA band and the shifted band of the protein-DNA complex are of equal intensity. From the conducted EMSA experiments, the K_D_ value of the MBP-RazD^HTH^ was estimated to be 0.7 to 0.8 μM. To narrow down the binding motif recognized by RazD in DNA fragment A, this DNA fragment was divided into smaller subfragments, A.1 (166 bp), A.2 (199 bp) and A.3 (102 bp), which were then also tested for binding of MBP-RazD^HTH^ in EMSA experiments. While DNA fragment A.2 retained the same band shift pattern (Fig. 4*E*) as the parental fragment A, indicating a K_D_ value of 0.7 to 0.8 μM, the two remaining DNA fragments A.1 (Fig. 4*D*) and A.3 (Fig. 4*F*) did not show any band shifts, suggesting that RazD is not binding these DNA sequences. Through shortening of the DNA fragments into fragment A.2.1 (116 bp, Fig. 4*G*) and A.2.2 (104 bp, Fig. 4*H*), the binding motif of MBP-RazD^HTH^ was further narrowed down to DNA fragment A.2.2, which was bound by the protein (Fig. 4*H*). This DNA fragment A.2.2 was then subdivided into eight DNA fragments with a length of 30 bp (Fig. 5*A*). When these DNA fragments were tested for binding of the MBP-RazD^HTH^ fusion protein, only for fragment A.2.2.7 a clear band shift was observed (Fig. 5*B*). We further divided A.2.2.7 into DNA fragments A.2.2.1 and A.2.2.2 (Fig. 5*A*). A.2.2.1 starts 10 bp upstream of the start of fragment A.2.2.7 and A.2.2.2 was also 10 bp longer at the 3′ end of A.2.2.1. We detected a weak band at the highest protein concentrations with A.2.2.2 but no shifted band with A.2.2.1 (Fig. 5*B*). This strongly suggests that the binding motif is located centrally within the 30 bp DNA fragment A.2.2.7. Furthermore, crucial elements of the binding motif are likely located within the 3′-half of DNA fragment A.2.2.7, which overlaps with the 5′-end of DNA fragment A.2.2.2 (Fig. 5*A*).

**Figure 5:**
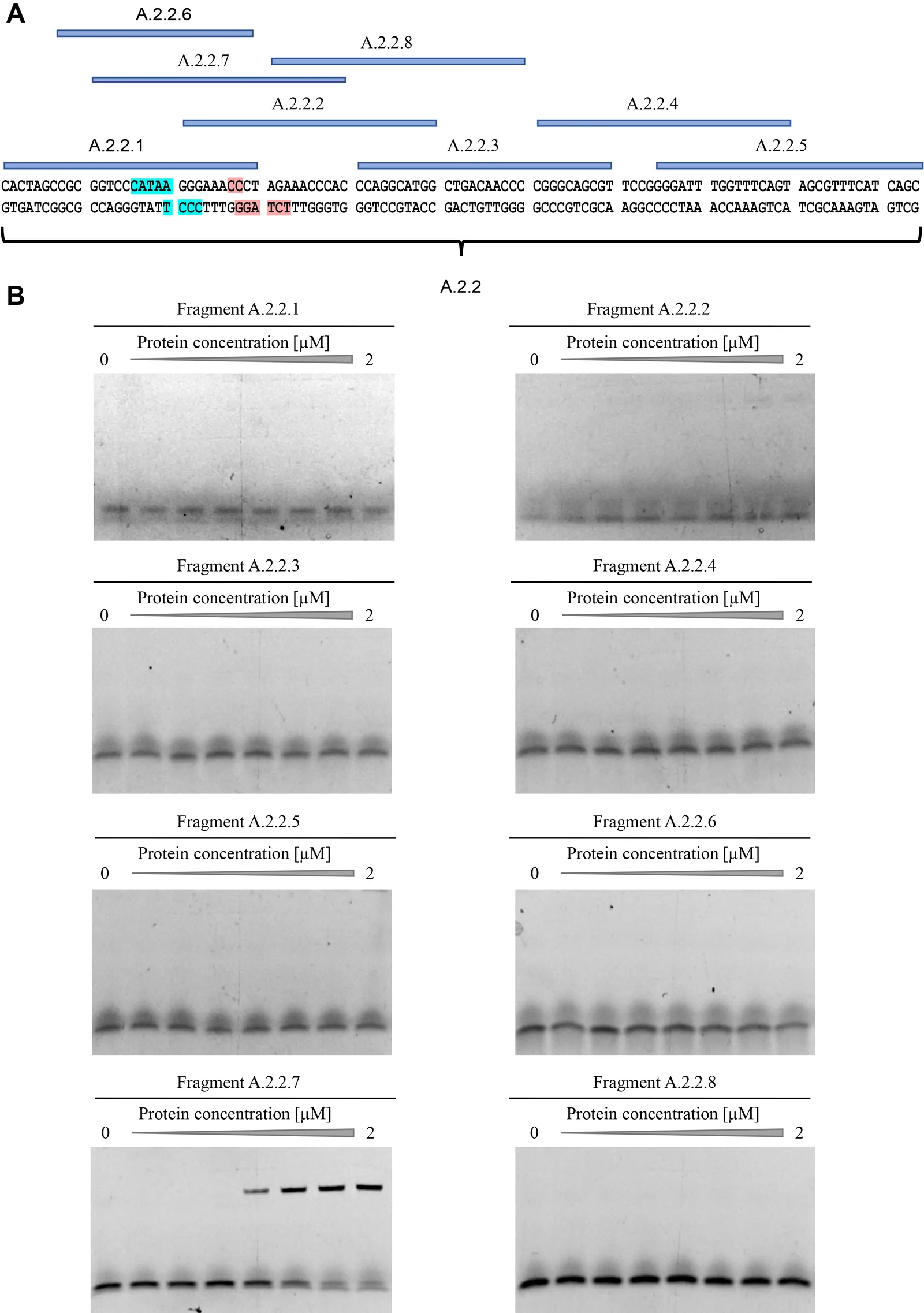
**Overview of the shortened DNA fragments used for EMSAs and the respective EMSAs.***A*, DNA fragment 2.2 covers the whole DNA sequence shown at the *bottom*. The shortened DNA fragments cover parts of this DNA sequence as indicated by the *blue lines*. DNA contact sites A (*red*) and B (*blue*) of RazD monomers are highlighted. *B*, EMSA using the shortened DNA fragments as probes. As expected due to the location of the DNA contact sites, except for DNA fragment A.2.2.7, none of the truncated DNA fragments showed binding to MBP-RazD^HTH^.

### Identification of the RazD DNA-binding motif

To characterize the binding site of RazD we used the AlphaFold 3 Server, a web-service that can generate highly accurate biomolecular structure predictions to model protein-DNA interactions based on the provided sequences (32). When modeling the binding of two RazD^HTH^ monomers to the DNA-fragment A.2.2.7, AlphaFold 3 predicted binding of the RazD HTH domain as a dimer (Fig. 6*A*). By uploading this structural model into the DNAproDB processing pipeline web server (33), the generated residue contact map shows individual nucleotide base-aa residue interactions of the two RazD^HTH^ protomers to the sequence 5′-TCTAGGGTTTCCCT-3′ of the coding (+) strand and to 5′-CATAAGGGAAACC-3′ of the complementary strand in the DNA fragment (Fig. 6*B*). Notably, all of the contacts between the α3 helices of both RazD^HTH^ protomers (Fig. 6*C*) are in the major groove of the DNA duplex.

**Figure 6:**
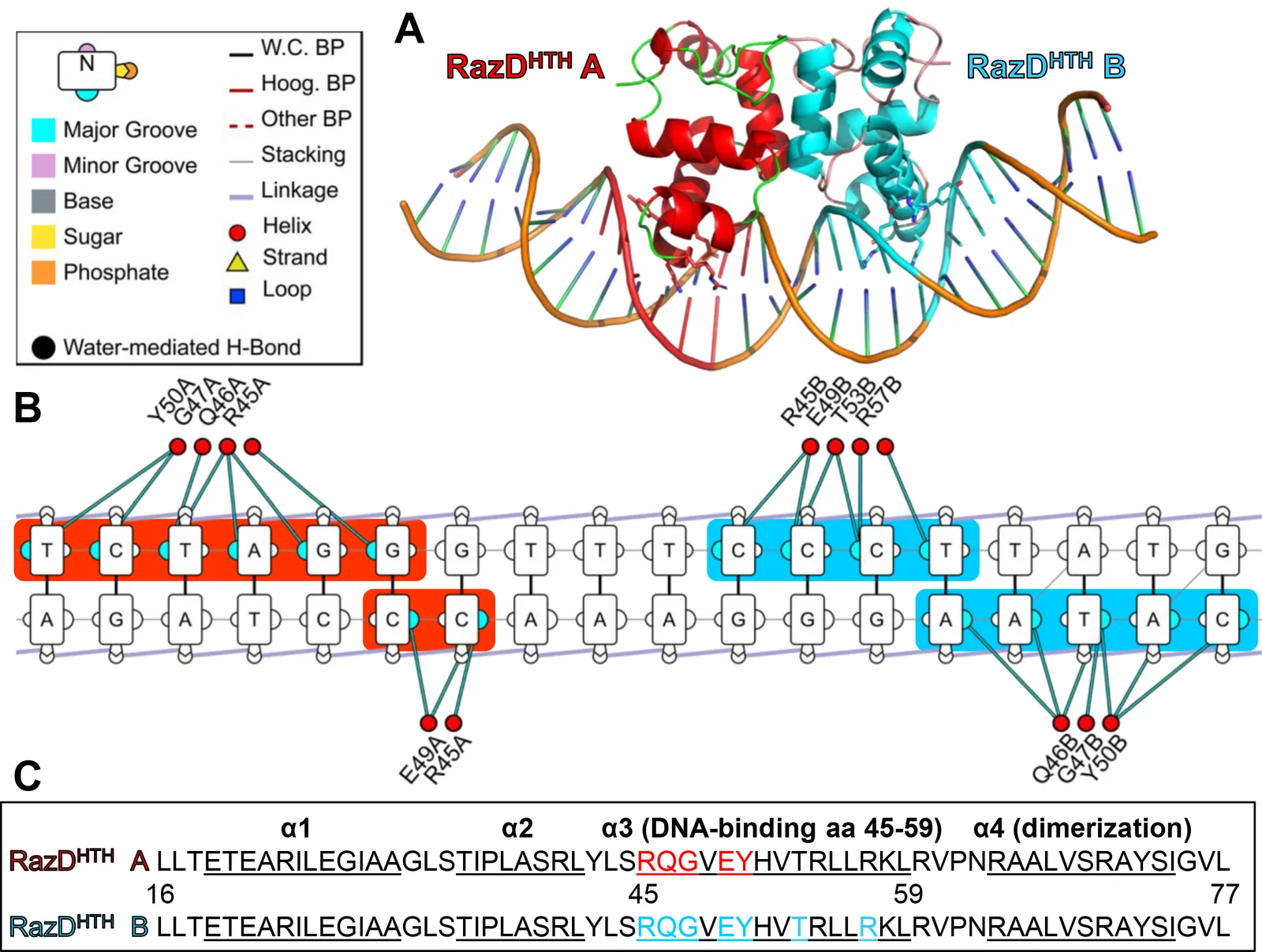
**AlphaFold 3 prediction of the protein:DNA complex structure of two RazDHTH monomers bound to its DNA-binding site in the *azlB* promoter.***A*, ribbon illustration of two RazD^HTH^ monomers (*red* and *blue*) bound to the 30 bp DNA duplex (A.2.2.7) derived from the *azlB* promoter. The confidence scores for the AlphaFold3 model are: ipTM (interface predicted template modeling score) = 0.84; pTM (predicted template modeling score) = 0.85. *B*, DNAproDB generated residue contact map visualizing the RazD^HTH^ aa side chain nucleotide base contacts of monomer A and B. *C*, residues of the C-terminal HTH domains A and B directly involved in specific DNA recognition are colored according to *panel A*. The secondary structure, including four α-helices is indicated at the top of the aa sequence.

To experimentally confirm the *azlB* DNA-binding site of RazD and to determine its binding kinetics, surface plasmon resonance (SPR) analyses were conducted (Fig. 7, *A*–*D*). For the SPR analysis, we used exactly the same DNA duplexes as used before for the EMSAs (Fig. 5). For DNA fragment A2.2.7, we observed high-affinity binding (K_D_ = 1.72 ± 0.05 nM) of the RazD^HTH^ domain (Fig. 7*B*), as observed before by EMSA (Fig. 5*B*). This finding further supports the hypothesis that the respective binding site of RazD is located within this 30 bp DNA fragment (Fig. 7*A*). Moreover, based on the immobilized DNA-amount that would yield a 50 RU maximum response (R_max_) upon binding of one RazD^HTH^ protomer, the measured R_max_ of 100 RU indicates cooperative DNA-binding of two RazD^HTH^ monomers. DNA fragments A.2.2.1 and A.2.2.2, which span a DNA region with a 20 bp overlap compared to fragment A.2.2.7, were also tested (Fig. 7*A*). As expected, DNA fragment A.2.2.1, in which the distal part of the predicted DNA binding site (5′-TCTAGG 3′) is only partially present and very close to the edge of the tested DNA fragment, lost high-affinity binding by the RazD^HTH^ domains (Fig. 7*C*), as also observed in EMSAs (Fig. 5*B*). Therefore, this DNA fragment served as a negative control for SPR analysis. Conversely, the measurements of DNA fragment A.2.2.2 revealed a moderate binding by the RazD^HTH^ domain (Fig. 7*D*) with an about 8-fold lower affinity compared to fragment A.2.2.7 (K_D_ = 13.4 ± 0.2 nM). The lower affinity of the RazD^HTH^ domain to DNA fragment A.2.2.2 may explain the very weak shifted band with this DNA fragment in the EMSA (Fig. 5*B*). In this DNA fragment A.2.2.2, the proximal RazD protomer B contact site 5′-CATAA-3′ is missing, while the distal RazD protomer A contact site (5′-TCTAGG-3′) is present.

**Figure 7:**
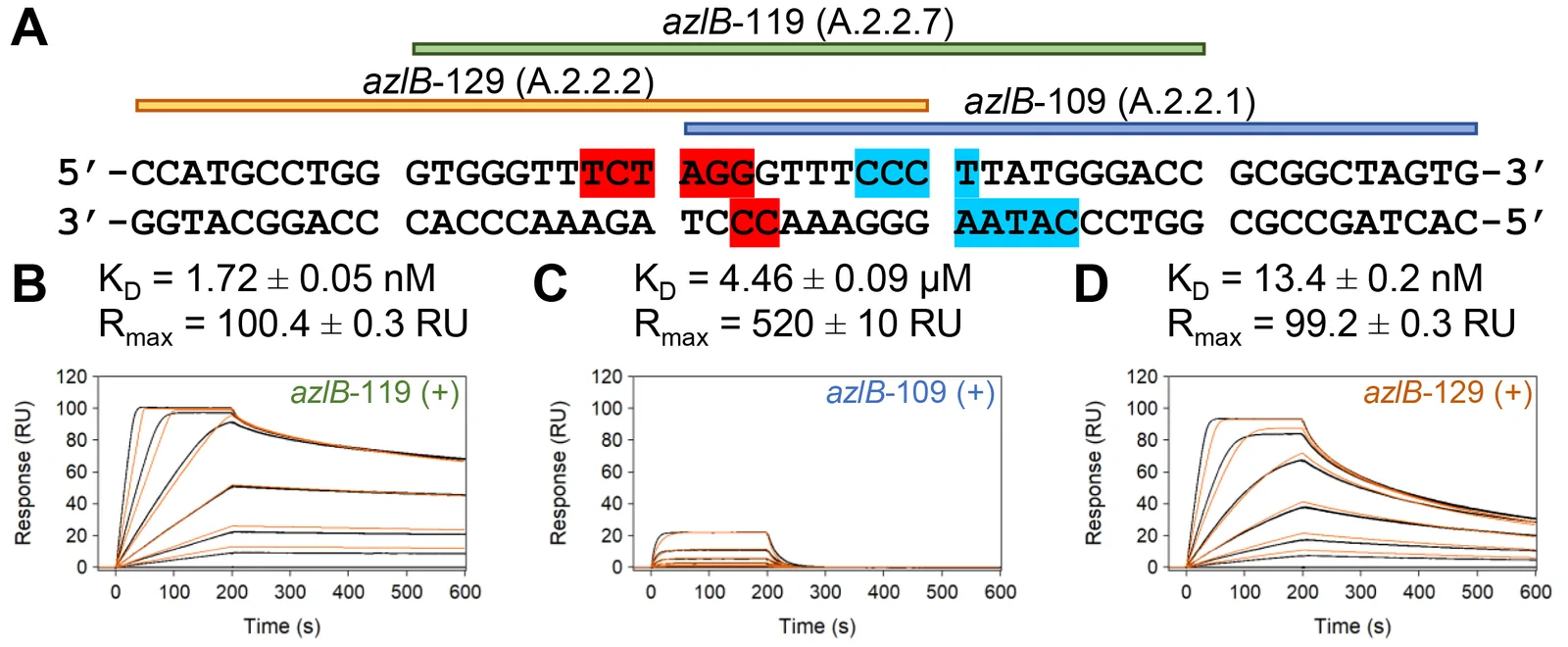
**Real-time *in vitro* surface plasmon resonance (SPR) binding analysis of RazDHTH to DNA duplexes.***A*, overview of the 30 bp DNA duplexes with predicted base contacts for the two RazDHTH protomers depicted in *red* and *blue*. The DNA sequences (*green*, *yellow*, *red lines*) used for SPR were derived from the upstream region of *azlB*, the first PKS gene of the *S. iranensis* azalomycin F BGC. They were exactly the same as used in the EMSA experiments shown in Figure 5*A*. The DNA fragments are located at positions. *B*, 119 (*azlB*-119; A.2.2.7), *C*) 109 (*azlB*-109; A.2.2.1) and *D*) 129 (*azlB*-129; A.2.2.2) upstream from the translational start site. Sensorgrams of 6.25, 12.5, 25, 50, 100 and 200 nM RazD^HTH^ binding injected in triplicate (*black lines*) are shown overlaid with the best fit derived from a 1:1 interaction model including a mass transport term (*red lines*). Measured dissociation constants (K_D_) and RazD^HTH^ maximum responses (R_max_) are plotted above the graphs.

Taken together, these results indicate that the complete RazD binding motif is indeed only located within DNA fragment A.2.2.7. Further, the analysis also showed that apparently the distal RazD protomer A contact nucleotides 5′-TCTAGG-3′ are most important for binding of a RazD dimer.

### Identification of RazD binding motifs in promoters of genes downregulated in the Δ*razD* mutant

After the identification of the DNA-binding motif of RazD in the upstream region of the first PKS of the azalomycin F BGC, we searched the literature and databases for LuxR-type regulators with similar DNA-binding motifs. Previously, Santos-Aberturas, Payero, Vicente, Guerra, Canibano, Martin, Aparicio (34) showed a functional conservation of PAS-LuxR transcriptional regulators across various *Streptomyces* spp., which produce polyene macrolides with antifungal activities. They discovered that the PimM regulator, which positively regulates the pimaricin BGC in *Streptomyces natalensis*, the NysRIV regulator, which activates nystatin biosynthesis in *Streptomyces noursei*, the AmphRIV regulator, which induces amphotericin B biosynthesis in *Streptomyces nodosus,* and PteF, an activator of the filipin biosynthesis in *Streptomyces avermitilis,* share not only the same regulatory pattern but also functionality. In addition, the authors (34) deduced the consensus sequence 5′-CTVGGGAWWTCCCBAG-3′ (V = not T, W = A or T, B = not A) for the DNA binding of these regulators based on EMSAs, DNase footprint assays, and bioinformatic analysis. Although this consensus sequence is shorter (16 bp), it shows high similarity to the RazD DNA-binding site upstream of *azlB* (*SIRAN_RS48655*, first PKS gene in the azalomycin F BGC) and its predicted protein-interacting nucleotides. Therefore, we added our binding site to the list of binding sites (Table S1) of the various PAS-LuxR transcriptional regulators published by Santos-Aberturas *et al.* (35) and fed this list into the MEME suite software version 5.8.8 (36) to adjust the original consensus sequence. The thus defined motif (5′-CTAGGGAWTTCCCGR-3′; W = A or T, R = A or G) was then fed into the FIMO software version 5.5.8 (Grant *et al.*, 2011) in order to search for further RazD DNA binding motifs in the promoter regions of a set of genes with at least 2-fold decreased expression in the *razD* deletion mutant (log2 fold changes < −1) compared to the wild type (Table S2).

We selected 10 promoters from this list with putative RazD-binding sites (E-value cutoff < 1e-04). Besides the already discovered site in the azalomycin F BGC, another binding site was predicted in the putative promoter region of *razD,* indicating that RazD is probably self-regulated. Additionally, one more binding site was predicted at the start of a butyrolactone BGC in the putative promoter region of *SIRAN_RS03185* (pyruvate phosphate dikinase). In addition, the putative promoter regions of two transporter systems (*SIRAN_RS13165* as well as *SIRAN_RS22710* and *SIRAN_RS22715,* which are probably co-transcribed) were predicted to encode a RazD-binding motif. Moreover, RazD-binding sites were predicted upstream of *SIRAN_RS26945* (metallopeptidase M64) and *SIRAN_RS30365* (LuxR-type regulator).

We tested in total 9 of the predicted binding sites by EMSAs to confirm binding of RazD. For predicted binding sites that were highly similar to the consensus sequence, we only used the predicted site in 30 bp DNA fragments; for less similar sequences, we tested DNA fragments containing the whole intergenic region. As a positive control (PC), without (−) and with (+) 2 μM MBP-RazD^HTH^ protein, we used the previously tested binding site upstream of *SIRAN_RS48655* (*azlB*) in the azalomycin F BGC and as a negative control (NC), again the unrelated gene *SIRAN_RS16895*. We observed binding of MBP-RazD^HTH^ to the 9 tested DNA fragments in the EMSAs (Table 2). Based on these results, we re-applied the MEME suite software version 5.8.8 (36) to adjust the originally used *Streptomyces* spp. consensus sequence specifically to *S. iranensis*. We thus propose 5′-CTAGGGAWTY-3′ (Y = C or T, W = A or T) as the minimal RazD DNA-binding motif for *S. iranensis* (Table 2), with 5′-CTAGG-3′ probably being the most important part of the binding motif, as predicted by AlphaFold 3 and experimentally shown by our SPR analysis.

**Table 2:** Overview of the Discovered RazD-Binding Sites (underlined) in Promoter Regions of Selected *S. iranensis* Genes With Decreased Expression in the *razD* Deletion Mutant

Regulated gene(s)	Putative gene function	EMSA	Positively tested EMSA probes	MEME motif and sites	Distance to putative gene start (strand localization)
*SIRAN_RS02585 SIRAN679*	*Streptomyces* antibiotic regulatory protein (SARP)		CCAGGACACTAGGTTTTCTGCCGGTGTTTT 30 bp	ACA CTAGGTTTTC TGC	−104 (+)
*SIRAN_RS03185 SIRAN805*	Pyruvate phosphate dikinase		GGCAATCGCTAGGGAAATGCGTAGCAACCA 30 bp	TCG CTAGGGAAAT GCG	−182 (+)
*azlB SIRAN_RS48655 SIRAN1043*	Polyketide synthase	Fragment A.2.2.7 (Fig. 5)	GTGGGTTTCTAGGGTTTCCCTTATGGGACC 30 bp	TTT CTAGGGTTTC CCT	−111 (+)
*razD SIRAN_RS04775 SIRAN1135*	LuxR-type PAS domain transcriptional regulator		Intergenic region, 270 bp	AGA CTAGGGATCT ACG	−188 (−)
*SIRAN_RS08520 SIRAN1904*	Protein arginine N-methyltransferase		TTCTATTCCTAGGGAATTCCGGGTTTTCGG 30 bp	TTC CTAGGGAATT CCG	−68 (+)
*SIRAN_RS13165 SIRAN2861*	Multidrug efflux transporter		Intergenic region, 434 bp	TCC TTAGGGATTT AAG	−9 (−)
*SIRAN_RS18590 SIRAN3976*	Hemolysin-type calcium-binding protein		GCGGGAAGCTAGGGGATTTCCGGAGCAACC 30 bp	AAG CTAGGGGATT TCC	−150 (+)
*SIRAN_RS22710-15 SIRAN4807-08*	ABC transporter		Intergenic region, 170 bp	AAG TTAAGGAACC ATA	−56 (−)
*SIRAN_RS26945 SIRAN5664*	Metallopeptidase M64		Intergenic region, 210 bp	GAA TTAGGGAAGT GGC	−94 (−)
*SIRAN_RS30365 SIRAN6363*	LuxR-type AAA ATPase domain transcriptional regulator		Intergenic region, 364 bp	CCA CTAGGGAAAC AAC	−597 (−)

For EMSAs, fluorescently labelled DNA probes from putative promoter regions were incubated with increasing amounts of the MBP-RazD^HTH^ fusion protein. As negative control (NC), the unrelated gene *SIRAN_RS16895* sequence was used either with (+) or without (−) MBP-RazD^HTH^. As positive control (PC), the upstream region of *SIRAN_RS48655/azlB* was used.

## Discussion

The interaction between microorganisms is driven by molecules, including NPs, that form the language of microorganisms (37, 38). An example is the arginoketide azalomycin F produced by *S. iranensis* and *S. rapamycinicus*. The compound activates the production of orsellinic acid and derivatives thereof in the fungus *A. nidulans* (11). For expression of the azalomycin F BGC, the regulatory gene *bldA* encoding tRNA^Leu^ is required (11). Based on the knowledge of *bldA,* here, we report the discovery of the regulatory island *razABCD*, which is not part of the azalomycin F BGC and is located about 120 kbp distant from the BGC. The *razABCD* regulatory island contains four genes encoding LuxR-family transcriptional regulators. While RazD is the main transcriptional activator of the BGC, both RazA and RazB are needed to activate the transcription of RazD (Fig. 8). It seems that RazD may also control the expression of *razABC* (modest repression), and that RazB may repress the expression of *razC*, and presumably itself given their genetic overlap. The function of RazC remains to be elucidated.

**Figure 8:**
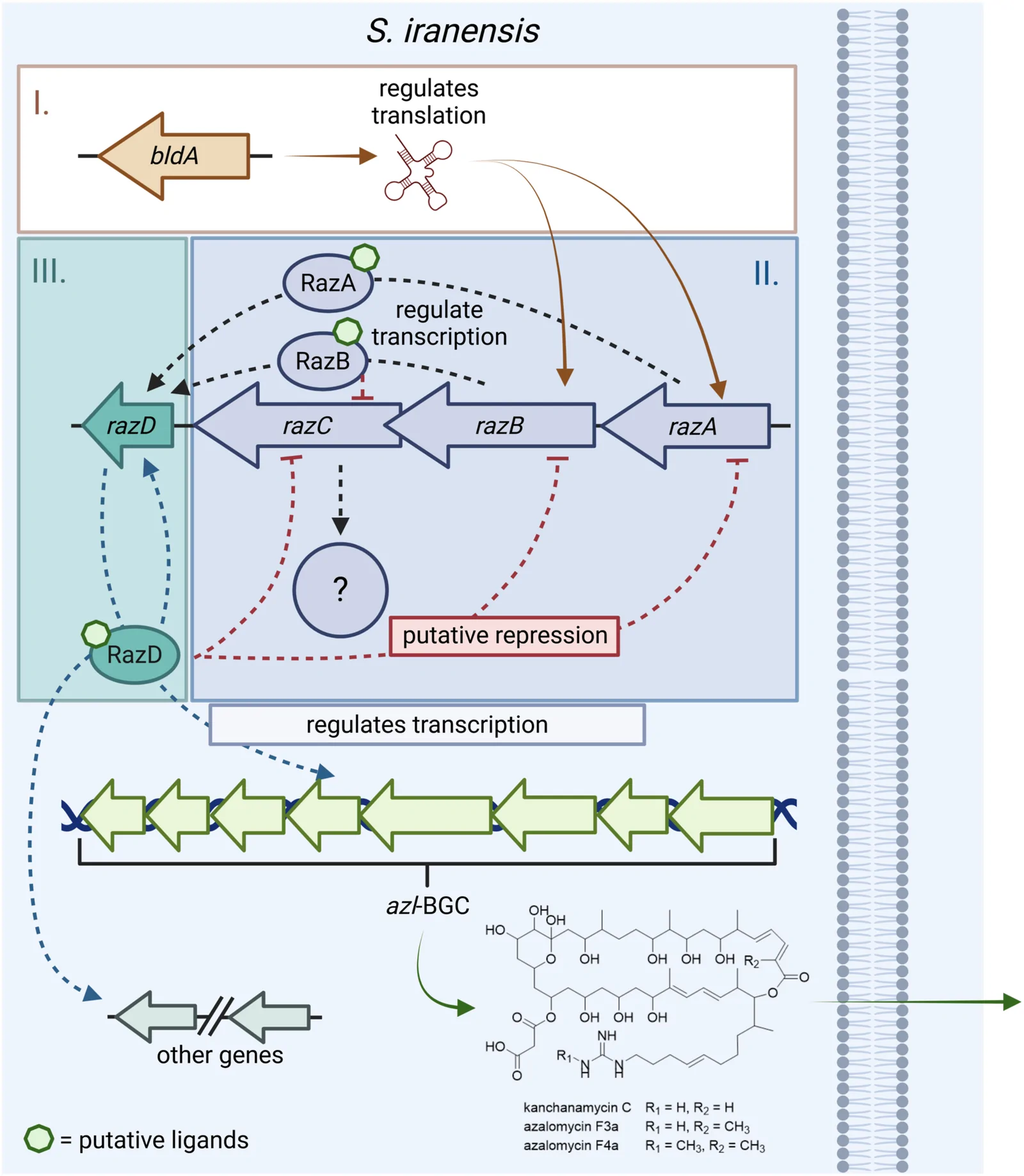
**Summary of the regulatory cascade leading to activation of the Azl BGC *via* the identified regulatory island *razABCD*.** The regulatory island *razABCD* regulates expression of the *azl*-BGC. RazAB constitute the second level of the regulatory hierarchy. RazB likely regulates expression of *razD* directly. RazD binds to the promoter region of the azl-BGC. The role of RacC has not been clarified yet. The indicated ligands of the LuxR-type transcription factors RazA-RazD are unknown. RazD may also control the expression of *razABC* (modest repression), and RazB may repress the expression of *razC*, and presumably itself, given their genetic overlap. RazD also activates genes not belonging to the *azl-*BGC like genes encoding an ABC transporter ATP-binding protein, a multidrug efflux transporter *etc.*

LuxR-type transcription factors typically contain a C-terminal helix-turn-helix (HTH) domain, responsible for DNA-binding, but vary in their N-terminal domain structure (39). Based on their domain architecture, these transcription factors can be sorted into different subgroups. RazD belongs to the PAS-LuxR-subtype whose members are rather small, usually between 200 and 400 aa long. In addition to their C-terminal HTH domain, they contain an N-terminal PAS (Per-Arnt-Sim) domain. PAS domains are capable of sensing a variety of signals that can be derived from intracellular processes or be provided extracellularly (40). Molecules sensed include oxygen, the redox state, and light, but also small molecules like cyclic nucleotides or carboxylic acids (41, 42, 43). At this point, it can only be speculated which ligands are bound by RazD and how this affects its regulatory functions.

According to AlphaFold 3, RazD is structurally similar to the PAS-LuxR transcription factor PimM, which positively regulates the pimaricin biosynthesis in *S. natalensis* (44). PimM shares its domain architecture and considerable sequence similarity with several other PAS-LuxR transcription factors regulating antifungal polyketide BGCs, namely AmphRIV (amphotericin), NysRIV (nystatin), FscRI (candicidin) and PteF (filipin) (44). Like RazD, NysIV of *S. noursei* is located on the chromosome as a member of four LuxR-type regulators with three large ATP-binding regulators of the LuxR family (LALs), such as RazA, RazB and RazC. These LALs usually comprise between 900 and 1000 aa and contain an N-terminal AAA (ATPases Associated with diverse cellular activities) domain in addition to their C-terminal HTH domain (45, 46). The AAA domain is usually responsible for binding and hydrolyzing ATP and thereby inducing a conformational change in the protein, affecting its DNA-binding capacity (29, 30). For the regulation of nystatin biosynthesis, Sekurova *et al.* (47) suggested a hierarchical order for the regulators and noticed that both *nysRII* (45% identity with RazB on aa sequence level) and *nysRIII* (53% identity with RazC on aa sequence level) were able to complement a Δ*nysRI* (62% identity with RazA on aa sequence level) mutant in cross-complementation experiments, but not each other. They assumed that they are both necessary for the expression of *nysRIV*, which in turn activates the transcription of the nystatin BGC, just like RazD does for the azalomycin F BGC. Here, the interaction between the LAL-type regulators appears to be different, since RazA and RazB are both necessary for the expression of the PAS-LuxR type regulator RazD, while RazC may be redundant although the latter remains speculative.

Thus, although there are many parallels in the activation of the azalomycin F and nystatin BGC, there are also many differences. While *nysRIV*, *pimM*, and *amphRIV* are located within their respective gene clusters, the group of LuxR-type regulators responsible for azalomycin F BGC activation is located 120 kbp distant from the BGC. According to AntiSMASH version 8.0.4 (48), the azalomycin F BGC contains four regulatory genes, the relevance of which awaits further elucidation. While NysRIV, PimM and AmphRIV have multiple DNA-binding sites within their respective gene clusters to control the expression of biosynthesis genes (35) as well as transporter-encoding genes, RazD seems to have only one binding site within the azalomycin F BGC. This might be due to the fact that the azalomycin F BGC is, in contrast to all the other BGCs, strictly unidirectionally organized. Thus, it might be transcribed from one promoter region, albeit the core biosynthesis genes span over 100 kbp. However, we also discovered binding sites spread throughout the genome, suggesting that the regulator controls regulatory circuits potentially connected to azalomycin F production. Also, RazD seems to control its own transcription, which Sekurova *et al.* (47) also suggested for NysRIV, as well as additional regulatory genes (*SIRAN_RS30365*), expanding the complex regulatory network even further. Additionally, RazD seems to activate the expression of two different transporter systems. Since the azalomycin F BGC does not contain any transport-related genes, one or both of them might be responsible for azalomycin F export. Similarly, a NysRIV binding site was discovered in the upstream region of NysH, a transporter located within the nystatin gene cluster (47). However, the azalomycin F BGC does not only lack putative transporter genes, but it also misses some of the genes necessary for the biosynthesis of azalomycin F3 and derivatives. Therefore, Hong *et al.* (13) assumed that, for example, in the azalomycin F producer *S*. *violaceusniger* DSM 4137 the genes necessary for generating the guanidinobutanoate starter unit might be shared between the azalomcycin F BGC and the desulfoclethramycin/clethramycin BGC. Interestingly, RazD also seems to activate transcription of a pyruvate phosphate dikinase (*SIRAN_RS03185*), which might be co-transcribed with a butyrolactone cluster and an M64 metallopeptidase (*SIRAN_RS26945*) that also contains an N-terminal signal sequence. However, whether these genes are connected to the azalomycin F biosynthesis remains to be shown.

## Experimental procedures

### Cultivation of microorganisms

*Escherichia coli* strains for cloning were grown at either 37 °C or 30 °C and 200 rpm in LB medium (Luria/Miller) (Carl Roth GmbH, Karlsruhe, Germany). The medium was supplemented with the respective antibiotics when necessary to maintain plasmids (Tables S3 and S4). For azalomycin F production, *S. iranensis* strains were inoculated (2 × 10^7^ spores) in 20 ml tryptic soy broth (CASO-bouillon, Carl Roth GmbH, Karlsruhe, Germany) in baffled Erlenmeyer flasks with silicone sponge lids and incubated at 28 °C with shaking (180 rpm) for 4 days to generate seeding cultures. When necessary to maintain plasmids, the respective antibiotics (Table S5) were added to the culture medium of mutants. Afterwards, a new culture (with 20 ml tryptic soy broth in baffled Erlenmeyer flasks) was inoculated with 2% of the seeding culture and incubated at 28 °C with shaking (180 rpm) on an orbital shaker for another 4 days without any antibiotics before the supernatant was sampled for HPLC-MS measurements. To generate spores for the experiments, 200 to 300 μl of densely grown cultures were streaked on oatmeal agar plates, which were incubated at 28 °C for 14 days before spores could be harvested.

### Generation of *S.* iranensis deletion plasmids

Gene deletions in *S. iranensis* were generated using homologous recombination and the temperature-sensitive vector pKOSi (49). For the generation of the deletion plasmid, the gene of interest as well as the 1 to 2 kb upstream and downstream regions of the gene were PCR amplified using primers listed (Table S6). The gene and its flanking regions were ligated into the vector pKOSi (49) using primers with attached restriction sites, and the generated plasmids were used for transformation of *E. coli* DH10β. In the next step, *E. coli* BW25113 pIJ790 was transformed with the newly generated pKOSi plasmid containing the gene to be deleted with its flanking region, and the gene to be deleted was replaced by the apramycin resistance cassette *aac(3)IV via* recombination using the λ Red system (50). For recombination, a PCR-amplified DNA fragment was used, which contained the *aac(3)IV* gene and an *oriT* amplified from pIJ790, as well as homologous sites at the ends generated by specific primers. The resulting plasmids were used to transform the non-methylating *E. coli* strain ET12567 pUZ8002, which was used for conjugation with *S. iranensis*.

### Generation of plasmids for complementation of deletion strains

For the generation of the complementation plasmids, the respective genes *razA*, *razB* and *razD* were PCR amplified using oligonucleotides (Table S6). The genes were ligated into the vector pIJ10257 (26) using restriction sites encoded by the oligonucleotides, and the yielded plasmids were then used to transform *E. coli* DH10β, from which the plasmids were re-isolated and checked for correctness. The correct plasmids pIJ *razA,* pIJ np_*razB*, and pIJ *razD* were then used for transformation of the non-methylating *E. coli* strain ET12567 pUZ8002 that was used for conjugation with *S. iranensis*.

### Intergeneric conjugation with *S. iranensis*

Conjugation was achieved either as described by Netzker *et al.* (49) or according to a modified protocol of Kieser *et al.* (51). Modifications of Kieser’s protocol include incubation of the heat-shocked spores for about 6 h at 28 °C before conjugation as well as using MS agar plates with 60 mM CaCl_2_ for conjugation, instead of MS agar with MgCl_2_.

### Detection of azalomycin F

To monitor azalomycin F production, *S. iranensis* wild type or mutant strains were grown in 100 ml TSB medium as described above for 5 days. Then, the cultures were extracted twice with a total of 100 ml ethyl acetate and concentrated under reduced pressure. For HPLC-MS analyses, the dried extracts were dissolved in 1 ml methanol, and 1 μl was loaded onto a LUNA^R^ Omega PS C18 LC-column with a particle size of 1.6 μm (Phenomenex) coupled to a Vanquish Flex UHPLC system equipped with a PDA and an Orbitrap Exploris 120 mass spectrometer (Thermo Fisher Scientific). As a solvent system water/acetonitrile were used both with 0.1% (v/v) formic acid and a flow rate of 0.5 ml/min (program over 13 min: initial 5% (v/v) acetonitrile hold for 0.5 min and increased to 70% (v/v) over 8 min held at 70% (v/v) for another 2 min and reversed to 5% (v/v) over 2 min.

### Transcriptome analysis

Total RNA of *S. iranensis* wild type and deletion mutants was isolated using the RNeasy Protect Bacteria Mini Kit (Qiagen GmbH, Hilden, Germany) according to the manufacturer’s instructions. Samples were taken after 48 h of cultivation in TSB medium as described above. Bacterial cells were disrupted by bead-beating using the ZR BashingBead Lysis Tubes 0.1 & 0.5 mm (Zymo Research Europe GmbH, Freiburg, Germany) and a FastPrep-24 5G (MP Biomedicals Germany GmbH) at 6.0 m/s for 60 s. After RNA purification with the RNeasy Protect Bacteria Mini Kit DNase treatment was performed using Baseline-ZERO DNase (Lucigen Corp), followed by a purification step using the RNeasy MinElute Cleanup Kit (Qiagen GmbH, Hilden, Germany). For each strain, total RNA from three replicates was analyzed. Library construction and paired-end 150 bp sequencing were performed by Novogene GmbH (Planegg).

### RNA-seq data processing

Preprocessing of raw reads including quality control and gene abundance estimation, was done with the GEO2RNaseq pipeline (v0.9.12; (52)) in R (version 3.5.1). Quality analysis was carried out with FastQC (v0.11.8) before and after trimming. Read-quality trimming was achieved with Trimmomatic (v0.36). Reads were rRNA-filtered using SortMeRNA (v2.1) with a single rRNA database combining all rRNA databases shipped with SortMeRNA. Reference annotation was created by extracting and combining exon features from corresponding annotation files. Reads were aligned to the NCBI reference genome of *S. iranensis* (assembly GCA_000938975.1) using HiSat2 (v2.1.0, paired-end mode). Gene abundance estimation was done with featureCounts (v1.28.0) in paired-end mode with default parameters. MultiQC version 1.7 was finally used to summarize and assess the quality of the output of FastQC, Trimmomatic, HiSat, featureCounts and SAMtools. The count matrix with gene abundance data without and with median-of-ratios normalization (MRN; (53)) was extracted. Differential gene expression was analyzed using GEO2RNaseq. Pairwise tests were performed using four statistical tools (DESeq v1.30.0, DESeq2 v1.18.1, limma voom v3.34.6 and edgeR v3.20.7) to report *p* values and multiple-testing-corrected *p* values using the false-discovery rate method q = FDR(*p*) for each tool. Gene expression differences were considered significant if they were reported significant by all four tools (q ≤ 0.05 and |log2 (MRN)| ≥ 1).

### Production and purification of recombinant RazD^HTH^

A codon-optimized synthetic gene (Thermo Fisher Scientific GeneArt) encoding the C-terminal helix-turn-helix (HTH) domain of *S. iranensis* RazD (residues 102–193, GenBank CDR03258.1) was cloned between the *Bam*HI/*Hin*dIII sites of a modified pET28a vector (Novagen Merck, Darmstadt, Germany). The resulting expression vector encodes RazD^HTH^ fused to the *E. coli* maltose-binding protein (MBP) lacking the leader aa sequence, *via* a polypeptide linker containing a hexahistidine tag followed by the tobacco etch virus (TEV) recognition site (ENLYFQ↓G). *E. coli* BL21(DE3) cells were transformed with the generated plasmid (New England Biolabs, Frankfurt, Germany). Cells were grown at 25 °C for 24 h in 1 L of auto-induction medium (Overnight Express Instant TB Medium; Novagen Merck), supplemented with 50 μg/ml kanamycin, harvested by centrifugation (10,500×*g*) and stored at −80 °C. Frozen cells (21 g) were resuspended in 200 ml buffer A (20 mM Tris/HCl, 200 mM NaCl, 1 mM EDTA, pH 7.5) containing 1 mM AEBSF and disrupted at 1000 bar using a high-pressure homogenizer (Emulsiflex C5, Avestin). After centrifugation (48,000×*g*) of the lysate, MBP-tagged RazD^HTH^ was purified by affinity chromatography using a 50 ml Dextrin Sepharose column (Cytiva Europe). The loaded Dextrin Sepharose column was washed with buffer A and MBP-tagged RazD^HTH^ was eluted with buffer A supplemented with 10 mM maltose. Pooled fractions were adjusted to 2 mM DTT and incubated overnight with recombinant TEV protease (made in-house, 4 μg TEV protease per mg MBP-RazD^HTH^) at room temperature.

The TEV-digested eluate was centrifuged with 8000×*g*, transferred to buffer B (50 mM NaH_2_PO_4_, pH 7.5) (HiPrep 26/10 Desalting column; Cytiva Europe) and loaded on a 5 ml HiTrap SP FF (Cytiva Europe, Freiburg, Germany) cation exchange chromatography (CIEX) column that was equilibrated with buffer B. The HiTrap SP FF column was washed with buffer B and eluted with a linear NaCl gradient (2 column volumes, up to 1 M NaCl) in buffer B. CIEX fractions containing RazD^HTH^ as well as MBP-RazD^HTH^ were further purified by size exclusion chromatography (SEC) on a Superdex prep-grade 75 16/60 column (Cytiva Europe) in 20 mM HEPES, 300 mM NaCl, 0.5 mM TCEP, pH 7.5. RazD^HTH^ and MBP-RazD^HTH^ protein preparations were snap-frozen in liquid N_2_ and stored at −80 °C.

### Electrophoretic mobility shift assay (EMSA)

EMSA experiments were performed according to Ream *et al.* (54) with slight modifications. For hybridization of DNA probes, the respective DNA oligonucleotides were mixed in an equimolar ratio, heated to 95 °C for 10 min and left at room temperature to cool down slowly. For protein-DNA binding reactions, protein aliquots were thawed on ice and then varying amounts of protein were mixed with 1 nM fluorescently labeled DNA probes, tenfold excess of salmon sperm DNA as competitor DNA (Invitrogen; Life Technologies, Darmstadt, Germany) as well as DNA binding buffer (10 × DNA binding buffer: 200 mM Tris-HCl, 100 mM NaCl, 10 mM EDTA, 10 mM DTT) in the required concentrations/amounts and filled-up to a reaction volume of 10 μl with ddH_2_O. The reaction solutions were mixed by finger-flicking and then incubated at room temperature for 1 h. Samples were run on agarose or polyacrylamide gels depending on the length of the analyzed DNA probes. For analysis of EMSA samples containing DNA probes of 80 bp or more, 2.5% (w/v) agarose gels in 0.5 x TB-buffer (45 mM TRIS, 45 mM boric acid, pH 7.5) were prepared according to (54). The 2.5% (w/v) agarose gels with a volume of 40 ml each were poured in gel slides, preheated to 60 °C. To each of the 10 μl EMSA reactions, 2 μl of 30% (v/v) glycerol was added for loading of the gel pockets. The gels were run at 200 V for 20 min (25 V/cm) in 0.5x TB buffer. To visualize the Cy3-labeled DNA probes, after electrophoresis, the gels were directly analyzed in a Fusion FX imaging device (Vilber Lourmat).

EMSA reactions using DNA probes below 80 bp in length were analyzed on polyacrylamide gels due to the better band resolution and separation properties offered by these gels. 5× high-density sample buffer (Novex^;^; Life Technologies) was added to the EMSA samples, and 10 μl of each sample were loaded on 6% (w/v) DNA Retardation Gels (Invitrogen; Life Technologies). The gels were run at 100 V for 20 min in 0.5 x TBE buffer (5 × Novex TBE buffer; 1:10 dilution) in an XCell *SureLock* Mini-cell (Invitrogen; Life Technologies, Darmstadt, Germany) and after electrophoresis, were directly analyzed in a Fusion FX imaging device (Vilber Lourmat).

### Surface plasmon resonance-based RazD^HTH^ DNA-binding assays

Real-time analyses were performed on a Biacore T200 system (Cytiva Europe) at 25 °C. DNA duplexes containing a putative RazD^HTH^ binding site from the 5′-upstream region of the *S. iranensis azlB* gene were produced by annealing complementary oligonucleotides using a fivefold molar excess of the non-biotinylated oligonucleotide. The dsDNA was injected at a flow rate of 10 μl/min onto flow cells 2, 3 and 4 of a streptavidin-coated SA sensor chip (Cytiva Europe) until the calculated amount of DNA was bound, giving a maximum RazD^HTH^ response of 100 RU after binding of two RazD^HTH^ monomers.

RazD^HTH^ samples were solved in running buffer (10 mM HEPES pH 7.4, 150 mM NaCl, 0.05% (v/v) surfactant P20, 0.5 mM TCEP, 10 μg/ml poly(dI-dC)) at concentrations of 6.25, 12.5, 25, 50, 100 and 200 nM (calculated for the monomer). Sample injection and dissociation times were set to 200 and 400 s at a flow rate of 30 μl/min. Each injection was performed three times. The chip surface was regenerated with 10 mM Tris/HCl pH 7.5, containing 0.5 M NaCl, 1 mM EDTA and 0.005% (w/v) SDS for 30 s. Refractive index errors due to bulk solvent effects were corrected with responses from DNA-free flow cell 1 as well as subtracting blank injections. Kinetic raw data were processed and globally fitted with Scrubber 2.0c (BioLogic Software) using a 1:1 interaction model including a mass transport term.

## Data availability

The RNA sequencing data presented in this paper are available from the ArrayExpress database (http://www.ebi.ac.uk/arrayexpress) under accession number E-MTAB-17219.

## Supporting information

This article contains supporting information: Figure S1 and Tables S1–S6.

## Conflict of interest

The authors declare that they have no conflicts of interest with the contents of this article.
