# Supplementary material for: The razABCD regulatory island links azalomycin F biosynthesis in Streptomyces iranensis to orsellinic acid production in the fungus Aspergillus nidulans
Source: J Biol Chem. 2026 Sep 18;302(11):113572. doi: 10.1016/j.jbc.2026.113572 (PMC13683440; doi:10.1016/j.jbc.2026.113572)
Supplement: Supplementary file 1 [file mmc1.docx]

**Supporting Information**

**The *razABCD* regulatory island links azalomycin F biosynthesis in *Streptomyces iranensis* to orsellinic acid production in the fungus *Aspergillus nidulans***

Lukas M Zehner^1, 2*^, Simone Edenhart^1*^, Peter Hortschansky^1^, Maira Rosin^1, 2^, Sascha Schäuble^3^, Gianni Panagiotou^2,3^, Tina Netzker^1^, Volker Schroeckh^1^, Axel A. Brakhage^1, 2^

**FIGURE S1.** Purification of recombinant *S. iranensis* RazD^HTH^. SDS-PAGE analysis of fractions from sequential dextrin affinity chromatography (DAC), cation exchange chromatography (CIEX) and size exclusion chromatography (SEC).

**TABLE S1.** PAS-LuxR regulatory protein binding sites according to Santos-Aberturas *et al*. (2011) with addition of the newly discovered *azlB* binding site site of RazD in *S. iranensis*.

| Promoter | Strand | Sequence |
| --- | --- | --- |
| *amphA*p | + | **TTAGGGAAAAGTACAG** |
| *amphA*p | - | **CTAAGGAATCTCAGTG** |
| *amphDI*p | + | **CTAGGGATTCCCCGGA** |
| *amphDI*p | - | **CCGGGGAATCCCTAGT** |
| *amphI*p | + | **CTAGGGATTTCCTGCC** |
| *amphI*p | - | **GGCAGGAAATCCCTAG** |
| *amphH*p | + | **CTAGGGAAATCCGGGT** |
| *amphH*p | - | **ACCCGGATTTCCCTAG** |
| *amphDIII*p | + | **CCCGGGAATTCCCTAG** |
| *amphDIII*p | - | **CTAGGGAATTCCCGGG** |
| *pteA1*p*_1* | + | **CTAGGGTTCTCCTTAG** |
| *pteA1*p*_1* | - | **TTAGGGGAATCCCCAA** |
| *pteA1*p*_2* | + | **TTGGGGATTCCCCTAA** |
| *pteA1*p*_2* | - | **CTAAGGAGAACCCTAG** |
| *nysA*p | + | **TTAGGGAAAAGTACAG** |
| *nysA*p | - | **CTAAGGAATGACGGGC** |
| *nysDI*p | + | **CTAGTGATTCCCCGGT** |
| *nysDI*p | - | **ACCGGGGAATCACTAG** |
| *nysI*p | + | **CTAGTGAATTCCTGCC** |
| *nysI*p | - | **GGCAGGAATTCACTAG** |
| *nysH*p | + | **CTAGTGAAATTTCGGA** |
| *nysH*p | - | **TCCGAAATTTCACTAG** |
| *nysDIII*p | + | **CTCGTGAATTCCCTAG** |
| *nysDIII*p | - | **CTAGGGAATTCACGAG** |
| *pimK*p | + | **CTAAGGATTTCCCGAC** |
| *pimK*p | - | **GTCGGGAAATCCTTAG** |
| *pimS2*p | + | **TCATGGAATTCCCTAG** |
| *pimS2*p | - | **CTAGGGAATTCCATGA** |
| *pimI*p | + | **CTAGGTGTTTTCCGGC** |
| *pimI*p | - | **GCCGGAAAACACCTAG** |
| *pimJ*p | + | **CCCGGAATTTCTTGAC** |
| *pimJ*p | - | **CTAGGGAATTCCCGAG** |
| *pimA*p | + | **ACCGGAAAACCCCTAG** |
| *pimA*p | - | **CTAGGGGTTTTCCGGT** |
| *pimE*p | + | **CTGGCATTTCCCCCAG** |
| *pimEp* | - | **GGGGGAAATGCCAGAG** |
| *pimS1*p | + | **CTGCGGGTTTCCCTAG** |
| *pimS1*p | - | **CTAGGGAAACCCGCAG** |
| *pimD*p | + | **GTAGGGATTTCCTGGT** |
| *pimD*p | - | **ACCAGGAAATCCCTAC** |
| *azlB*p | + | **TCTAGGGTTTCCCTTATG** |
| *azlB*p | - | **CATAAGGGAAACCCTAGA** |
|  | MEME motif |  |

**TABLE S2. List of genes with decreased (red) and increased (blue) expression in the *S. iranensis ΔrazD* mutant compared to the wild type.** Listed are only genes with log2 fold changes > 1 or < -1 and p-value of 1E-04.

| **Locus tag**  **NZ_LK022848.1** | **Locus tag**  **LK022848.1** | **Gene** | **Gene function** | **Δ*razD* vs. WT**  **log2 rpkm** | **DESeq2 adjusted**  **p-value** |
| --- | --- | --- | --- | --- | --- |
| *SIRAN_RS04775* | SIRAN1135 | *razD* | PAS-LuxR regulator | -8.87 | 4.13E-43 |
| *SIRAN_RS15755* | SIRAN3381 |  | Integral membrane protein | -7.79 | 0.00E+00 |
| *SIRAN_RS52360* |  | *azlE4* | Azalomycin F BGC | -6.88 | 0.00E+00 |
| *SIRAN_RS04315* |  | *azlB1* |  | -6.88 | 0.00E+00 |
| *SIRAN_RS52355* |  | *azlE3* |  | -6.75 | 0.00E+00 |
| *SIRAN_RS52350* |  | *azlE2* |  | -6.65 | 0.00E+00 |
| *SIRAN_RS49975* |  | *azlC* |  | -6.57 | 2.85E-296 |
| *SIRAN_RS48635* |  | *azlE1* |  | -6.55 | 0.00E+00 |
| *SIRAN_RS52345* |  | *azlF2* |  | -6.52 | 0.00E+00 |
| *SIRAN_RS04265* |  | *azlF1* |  | -6.36 | 0.00E+00 |
| *SIRAN_RS04325* |  | *azl3* |  | -6.36 | 1.21E-37 |
| *SIRAN_RS52370* |  | *azlD2* |  | -6.28 | 9.33E-282 |
| *SIRAN_RS52365* |  | *azlD1* |  | -6.19 | 9.84E-255 |
| *SIRAN_RS48655* | SIRAN1034 | *azlB2* |  | -6.18 | 1.86E-223 |
| *SIRAN_RS04250* |  | *azlH* |  | -5.98 | 0.00E+00 |
| *SIRAN_RS04260* |  | *azlG2* |  | -5.95 | 0.00E+00 |
| *SIRAN_RS04255* |  | *azlG1* |  | -5.86 | 6.64E-217 |
| *SIRAN_RS04245* |  | *azlA* |  | -5.71 | 0.00E+00 |
| *SIRAN_RS04330* |  | *azl2* |  | -5.67 | 1.88E-126 |
| *SIRAN_RS04240* |  | *azl4* |  | -5.51 | 4.07E-204 |
| *SIRAN_RS04235* |  | *azl5* |  | -5.37 | 9.29E-209 |
| *SIRAN_RS15760* | SIRAN3382 |  | Histidine kinase | -4.49 | 3.39E-106 |
| *SIRAN_RS33905* | SIRAN7088 |  | ABC transporter permease | -4.48 | 3.13E-133 |
| *SIRAN_RS33910* | SIRAN7089 |  | ATP-binding cassette domain-containing protein | -4 | 2.23E-133 |
| *SIRAN_RS27975* | SIRAN5878 |  | ABC transporter ATP-binding protein | -3.96 | 6.77E-16 |
| *SIRAN_RS27970* | SIRAN5877 |  | ABC transporter permease | -3.84 | 4.99E-10 |
| *SIRAN_RS22710* | SIRAN4807 |  | ABC transporter | -3.47 | 1.02E-10 |
| *SIRAN_RS13165* | SIRAN2861 |  | Multidrug efflux transporter | -2.92 | 8.06E-05 |
| *SIRAN_RS24560* | SIRAN5182 |  | SigE family RNA polymerase sigma factor | -2.92 | 5.59E-44 |
| *SIRAN_RS20480* | SIRAN4370 |  | Acyltransferase | -2.91 | 1.79E-44 |
| *SIRAN_RS15765* | SIRAN3383 |  | LuxR family response regulator | -2.83 | 1.28E-54 |
| *SIRAN_RS02585* | SIRAN679 |  | SARP family transcriptional activator | -2.46 | 2.97E-45 |
| *SIRAN_RS22715* | SIRAN4808 |  | ABC transporter | -2.42 | 1.97E-23 |
| *SIRAN_RS06245* | SIRAN1437 |  | Drug/metabolite transporter | -2.39 | 1.23E-30 |
| *SIRAN_RS30360* | SIRAN6362 |  | Flavin monoamine oxidase | -2.38 | 3.74E-55 |
| *SIRAN_RS08060* |  |  |  | -2.16 | 1.57E-26 |
| *SIRAN_RS53350* |  |  |  | -2.06 | 9.65E-18 |
| *SIRAN_RS47035* |  |  |  | -2.03 | 6.49E-14 |
| *SIRAN_RS13170* |  |  |  | -2.01 | 4.98E-04 |
| *SIRAN_RS04230* |  |  |  | -1.94 | 2.79E-32 |
| *SIRAN_RS47010* |  |  |  | -1.91 | 9.64E-18 |
| *SIRAN_RS26945* | SIRAN5664 |  | Metallopeptidase M64 | -1.9 | 1.20E-23 |
| *SIRAN_RS06250* |  |  |  | -1.88 | 3.14E-14 |
| *SIRAN_RS08055* |  |  |  | -1.87 | 2.35E-17 |
| *SIRAN_RS02605* |  |  |  | -1.83 | 3.50E-10 |
| *SIRAN_RS47015* |  |  |  | -1.8 | 9.87E-11 |
| *SIRAN_RS47000* |  |  |  | -1.77 | 3.79E-14 |
| *SIRAN_RS50145* |  |  |  | -1.74 | 1.57E-14 |
| *SIRAN_RS47030* |  |  |  | -1.66 | 2.16E-10 |
| *SIRAN_RS47025* |  |  |  | -1.66 | 2.96E-11 |
| *SIRAN_RS03190* |  |  |  | -1.57 | 1.58E-12 |
| *SIRAN_RS25820* |  |  |  | -1.53 | 1.21E-18 |
| *SIRAN_RS08070* |  |  |  | -1.51 | 8.17E-10 |
| *SIRAN_RS46995* |  |  |  | -1.48 | 2.47E-10 |
| *SIRAN_RS22705* |  |  |  | -1.45 | 9.35E-12 |
| *SIRAN_RS08045* |  |  |  | -1.41 | 1.56E-18 |
| *SIRAN_RS46990* |  |  |  | -1.41 | 1.35E-08 |
| *SIRAN_RS01710* |  |  |  | -1.41 | 1.16E-10 |
| *SIRAN_RS15745* |  |  |  | -1.37 | 5.36E-07 |
| *SIRAN_RS02600* |  |  |  | -1.36 | 4.00E-11 |
| *SIRAN_RS30355* | SIRAN6361 |  | AMP-dependent synthetase and ligase | -1.32 | 3.16E-08 |
| *SIRAN_RS51805* |  |  |  | -1.32 | 1.06E-33 |
| *SIRAN_RS30365* | SIRAN6363 |  | LuxR family transcriptional regulator | -1.31 | 7.65E-18 |
| *SIRAN_RS23265* |  |  |  | -1.27 | 2.00E-05 |
| *SIRAN_RS03185* | SIRAN805 |  | Pyruvate phosphate dikinase | -1.25 | 2.02E-20 |
| *SIRAN_RS03180* |  |  |  | -1.22 | 4.01E-22 |
| *SIRAN_RS08515* |  |  |  | -1.21 | 1.21E-15 |
| *SIRAN_RS09835* |  |  |  | -1.2 | 1.57E-11 |
| *SIRAN_RS13120* |  |  |  | -1.15 | 5.28E-06 |
| *SIRAN_RS43090* |  |  |  | -1.14 | 6.77E-19 |
| *SIRAN_RS03130* |  |  |  | -1.14 | 4.86E-14 |
| *SIRAN_RS08050* |  |  |  | -1.13 | 4.84E-13 |
| *SIRAN_RS05090* |  |  |  | -1.13 | 2.07E-09 |
| *SIRAN_RS15750* |  |  |  | -1.12 | 3.50E-11 |
| *SIRAN_RS08520* | SIRAN1904 |  | Protein arginine N-methyltransferase | -1.11 | 5.73E-18 |
| *SIRAN_RS32235* |  |  |  | -1.08 | 4.16E-09 |
| *SIRAN_RS43015* |  |  |  | -1.08 | 3.84E-06 |
| *SIRAN_RS41890* |  |  |  | -1.07 | 1.51E-10 |
| *SIRAN_RS25825* |  |  |  | -1.06 | 1.11E-08 |
| *SIRAN_RS03175* |  |  |  | -1.06 | 2.23E-18 |
| *SIRAN_RS37335* |  |  |  | -1.05 | 4.39E-17 |
| *SIRAN_RS03135* |  |  |  | -1.04 | 2.75E-13 |
| *SIRAN_RS52885* |  |  |  | -1.04 | 4.23E-09 |
| *SIRAN_RS28340* |  |  |  | -1.01 | 3.76E-08 |
| *SIRAN_RS18590* | SIRAN3976 |  | Hemolysin-type calcium-binding protein | -1.01 | 8.07E-09 |
| *SIRAN_RS29405* |  |  |  | 1.00 | 9.68E-06 |
| *SIRAN_RS15465* |  |  |  | 1.01 | 1.38E-04 |
| *SIRAN_RS45205* |  |  |  | 1.01 | 9.76E-07 |
| *SIRAN_RS06960* |  |  |  | 1.01 | 6.19E-04 |
| *SIRAN_RS36655* |  |  |  | 1.02 | 4.71E-05 |
| *SIRAN_RS21220* |  |  |  | 1.02 | 9.54E-05 |
| *SIRAN_RS44205* |  |  |  | 1.02 | 3.82E-04 |
| *SIRAN_RS44175* |  |  |  | 1.03 | 4.02E-09 |
| *SIRAN_RS38710* |  |  |  | 1.03 | 1.68E-08 |
| *SIRAN_RS53235* |  |  |  | 1.03 | 1.42E-06 |
| *SIRAN_RS35095* |  |  |  | 1.03 | 2.63E-04 |
| *SIRAN_RS27250* |  |  |  | 1.03 | 2.67E-06 |
| *SIRAN_RS15455* |  |  |  | 1.04 | 2.21E-04 |
| *SIRAN_RS07645* |  |  |  | 1.04 | 1.97E-04 |
| *SIRAN_RS47715* |  |  |  | 1.05 | 9.73E-05 |
| *SIRAN_RS26505* |  |  |  | 1.06 | 3.47E-09 |
| *SIRAN_RS12925* |  |  |  | 1.06 | 1.79E-05 |
| *SIRAN_RS29935* |  |  |  | 1.06 | 7.12E-04 |
| *SIRAN_RS41390* |  |  |  | 1.06 | 2.06E-05 |
| *SIRAN_RS38585* |  |  |  | 1.06 | 7.26E-06 |
| *SIRAN_RS15670* |  |  |  | 1.06 | 1.70E-05 |
| *SIRAN_RS31555* |  |  |  | 1.07 | 7.22E-06 |
| *SIRAN_RS51850* |  |  |  | 1.07 | 1.36E-06 |
| *SIRAN_RS29480* |  |  |  | 1.07 | 1.55E-05 |
| *SIRAN_RS36590* |  |  |  | 1.07 | 2.41E-04 |
| *SIRAN_RS06600* |  |  |  | 1.07 | 2.45E-06 |
| *SIRAN_RS18920* |  |  |  | 1.08 | 3.28E-07 |
| *SIRAN_RS35325* |  |  |  | 1.09 | 5.15E-04 |
| *SIRAN_RS06980* |  |  |  | 1.09 | 1.87E-04 |
| *SIRAN_RS38655* |  |  |  | 1.09 | 6.33E-05 |
| *SIRAN_RS45175* |  |  |  | 1.09 | 9.73E-04 |
| *SIRAN_RS12930* |  |  |  | 1.10 | 1.05E-04 |
| *SIRAN_RS53245* |  |  |  | 1.10 | 4.86E-05 |
| *SIRAN_RS38575* |  |  |  | 1.10 | 3.86E-06 |
| *SIRAN_RS05995* |  |  |  | 1.10 | 1.59E-04 |
| *SIRAN_RS00030* |  |  |  | 1.11 | 2.55E-05 |
| *SIRAN_RS44890* |  |  |  | 1.11 | 3.14E-04 |
| *SIRAN_RS36595* |  |  |  | 1.12 | 4.01E-04 |
| *SIRAN_RS43850* |  |  |  | 1.12 | 3.69E-06 |
| *SIRAN_RS38595* |  |  |  | 1.12 | 3.17E-09 |
| *SIRAN_RS44130* |  |  |  | 1.13 | 6.17E-05 |
| *SIRAN_RS38590* |  |  |  | 1.13 | 6.42E-06 |
| *SIRAN_RS05020* |  |  |  | 1.14 | 1.79E-07 |
| *SIRAN_RS06010* |  |  |  | 1.14 | 7.92E-05 |
| *SIRAN_RS36600* |  |  |  | 1.15 | 5.16E-06 |
| *SIRAN_RS52880* |  |  |  | 1.15 | 3.70E-05 |
| *SIRAN_RS42735* |  |  |  | 1.15 | 3.32E-04 |
| *SIRAN_RS44120* |  |  |  | 1.16 | 2.93E-05 |
| *SIRAN_RS38630* |  |  |  | 1.16 | 7.78E-05 |
| *SIRAN_RS38510* |  |  |  | 1.16 | 1.07E-08 |
| *SIRAN_RS21930* |  |  |  | 1.16 | 2.41E-05 |
| *SIRAN_RS44415* |  |  |  | 1.17 | 9.08E-04 |
| *SIRAN_RS28100* |  |  |  | 1.18 | 5.37E-04 |
| *SIRAN_RS49670* |  |  |  | 1.18 | 2.47E-13 |
| *SIRAN_RS38635* |  |  |  | 1.18 | 1.53E-04 |
| *SIRAN_RS14720* |  |  |  | 1.19 | 1.55E-10 |
| *SIRAN_RS38570* |  |  |  | 1.19 | 1.10E-08 |
| *SIRAN_RS53240* |  |  |  | 1.19 | 1.78E-05 |
| *SIRAN_RS06975* |  |  |  | 1.19 | 2.85E-07 |
| *SIRAN_RS38650* |  |  |  | 1.19 | 5.99E-09 |
| *SIRAN_RS08935* |  |  |  | 1.20 | 1.04E-06 |
| *SIRAN_RS05160* |  |  |  | 1.21 | 1.70E-06 |
| *SIRAN_RS36650* |  |  |  | 1.21 | 2.31E-04 |
| *SIRAN_RS38600* |  |  |  | 1.21 | 1.10E-08 |
| *SIRAN_RS38615* |  |  |  | 1.22 | 4.97E-05 |
| *SIRAN_RS00035* |  |  |  | 1.22 | 6.91E-06 |
| *SIRAN_RS35400* |  |  |  | 1.22 | 3.69E-05 |
| *SIRAN_RS38660* |  |  |  | 1.23 | 4.68E-07 |
| *SIRAN_RS38625* |  |  |  | 1.23 | 3.39E-07 |
| *SIRAN_RS33015* |  |  |  | 1.23 | 1.31E-04 |
| *SIRAN_RS06595* |  |  |  | 1.23 | 1.63E-08 |
| *SIRAN_RS17230* |  |  |  | 1.23 | 1.19E-06 |
| *SIRAN_RS18055* |  |  |  | 1.24 | 4.44E-07 |
| *SIRAN_RS38580* |  |  |  | 1.24 | 5.62E-09 |
| *SIRAN_RS12935* |  |  |  | 1.24 | 3.23E-05 |
| *SIRAN_RS38605* |  |  |  | 1.25 | 2.09E-08 |
| *SIRAN_RS19285* |  |  |  | 1.25 | 4.61E-12 |
| *SIRAN_RS38705* |  |  |  | 1.26 | 5.02E-09 |
| *SIRAN_RS51855* |  |  |  | 1.26 | 4.10E-07 |
| *SIRAN_RS04630* |  |  |  | 1.26 | 3.57E-05 |
| *SIRAN_RS07650* |  |  |  | 1.27 | 1.39E-04 |
| *SIRAN_RS46135* |  |  |  | 1.27 | 2.84E-06 |
| *SIRAN_RS00100* |  |  |  | 1.27 | 4.80E-11 |
| *SIRAN_RS38620* |  |  |  | 1.28 | 1.68E-08 |
| *SIRAN_RS08810* |  |  |  | 1.29 | 5.42E-10 |
| *SIRAN_RS52200* |  |  |  | 1.30 | 1.78E-08 |
| *SIRAN_RS52190* |  |  |  | 1.31 | 8.25E-09 |
| *SIRAN_RS45070* |  |  |  | 1.31 | 5.64E-06 |
| *SIRAN_RS41395* |  |  |  | 1.32 | 6.82E-07 |
| *SIRAN_RS38610* |  |  |  | 1.33 | 1.39E-08 |
| *SIRAN_RS41400* |  |  |  | 1.34 | 2.34E-08 |
| *SIRAN_RS00110* |  |  |  | 1.34 | 7.12E-10 |
| *SIRAN_RS38465* |  |  |  | 1.34 | 2.9E-08 |
| *SIRAN_RS26880* |  |  |  | 1.35 | 2.97E-08 |
| *SIRAN_RS03455* |  |  |  | 1.35 | 7.21E-07 |
| *SIRAN_RS46170* |  |  |  | 1.35 | 3.63E-10 |
| *SIRAN_RS52180* |  |  |  | 1.35 | 7.44E-13 |
| *SIRAN_RS38645* |  |  |  | 1.35 | 2.92E-09 |
| *SIRAN_RS32930* |  |  |  | 1.36 | 6.40E-10 |
| *SIRAN_RS38640* |  |  |  | 1.36 | 7.13E-07 |
| *SIRAN_RS04625* |  |  |  | 1.36 | 2.16E-05 |
| *SIRAN_RS04640* |  |  |  | 1.38 | 1.24E-06 |
| *SIRAN_RS04635* |  |  |  | 1.39 | 3.79E-06 |
| *SIRAN_RS48495* |  |  |  | 1.39 | 1.44E-13 |
| *SIRAN_RS51870* |  |  |  | 1.40 | 1.42E-05 |
| *SIRAN_RS51845* |  |  |  | 1.42 | 5.43E-10 |
| *SIRAN_RS00045* |  |  |  | 1.42 | 8.53E-10 |
| *SIRAN_RS45210* |  |  |  | 1.43 | 8.10E-13 |
| *SIRAN_RS27800* |  |  |  | 1.46 | 2.83E-08 |
| *SIRAN_RS45215* |  |  |  | 1.48 | 3.00E-12 |
| *SIRAN_RS00055* |  |  |  | 1.48 | 3.88E-14 |
| *SIRAN_RS18240* |  |  |  | 1.48 | 1.92E-04 |
| *SIRAN_RS31520* |  |  |  | 1.49 | 8.96E-11 |
| *SIRAN_RS52195* |  |  |  | 1.49 | 6.83E-09 |
| *SIRAN_RS18225* |  |  |  | 1.50 | 1.18E-07 |
| *SIRAN_RS06945* |  |  |  | 1.50 | 9.20E-07 |
| *SIRAN_RS00065* |  |  |  | 1.50 | 9.12E-15 |
| *SIRAN_RS48500* |  |  |  | 1.51 | 3.12E-19 |
| *SIRAN_RS08945* |  |  |  | 1.51 | 4.37E-07 |
| *SIRAN_RS00080* |  |  |  | 1.52 | 6.98E-15 |
| *SIRAN_RS51590* |  |  |  | 1.53 | 5.56E-11 |
| *SIRAN_RS00075* |  |  |  | 1.53 | 1.34E-14 |
| *SIRAN_RS00095* |  |  |  | 1.57 | 2.41E-12 |
| *SIRAN_RS00040* |  |  |  | 1.57 | 2.40E-08 |
| *SIRAN_RS44170* |  |  |  | 1.63 | 2.52E-25 |
| *SIRAN_RS53185* |  |  |  | 1.64 | 9.64E-18 |
| *SIRAN_RS00090* |  |  |  | 1.65 | 4.04E-15 |
| *SIRAN_RS44180* |  |  |  | 1.68 | 7.99E-14 |
| *SIRAN_RS44195* |  |  |  | 1.69 | 6.01E-15 |
| *SIRAN_RS47215* |  |  |  | 1.70 | 1.31E-15 |
| *SIRAN_RS38700* |  |  |  | 1.71 | 5.65E-14 |
| *SIRAN_RS44135* |  |  |  | 1.73 | 5.61E-14 |
| *SIRAN_RS04645* |  |  |  | 1.74 | 3.47E-09 |
| *SIRAN_RS00070* |  |  |  | 1.74 | 1.21E-13 |
| *SIRAN_RS44140* |  |  |  | 1.77 | 1.59E-23 |
| *SIRAN_RS34565* |  |  |  | 1.78 | 7.90E-10 |
| *SIRAN_RS31525* |  |  |  | 1.83 | 8.02E-18 |
| *SIRAN_RS44145* |  |  |  | 1.86 | 2.48E-34 |
| *SIRAN_RS46140* |  |  |  | 1.93 | 1.17E-10 |
| *SIRAN_RS44190* |  |  |  | 2.03 | 8.24E-07 |
| *SIRAN_RS46150* |  |  |  | 2.04 | 1.08E-22 |
| *SIRAN_RS44165* |  |  |  | 2.22 | 1.08E-22 |
| *SIRAN_RS51860* |  |  |  | 2.27 | 1.40E-30 |
| *SIRAN_RS47200* |  |  |  | 2.92 | 2.63E-36 |
| *SIRAN_RS47210* |  |  |  | 3.07 | 3.90E-14 |
| *SIRAN_RS46160* |  |  |  | 3.08 | 1.54E-29 |
| *SIRAN_RS47205* |  |  |  | 3.57 | 2.04E-13 |
| *SIRAN_RS46155* |  |  |  | 3.68 | 7.53E-08 |
| *SIRAN_RS51975* |  |  |  | 3.74 | 8.82E-63 |
| *SIRAN_RS46145* |  |  |  | 3.80 | 4.82E-44 |
| *SIRAN_RS46165* |  |  |  | 3.84 | 5.96E-08 |
| *SIRAN_RS51970* |  |  |  | 4.00 | 3.52E-09 |

**TABLE S3** Bacterial strains used in this study.

| Strain | Relevant features | Reference |
| --- | --- | --- |
| *Streptomyces iranensis* DSM41954 (HM35^T^) | Wild-type strain | Hamedi *et al*., 2010 |
| *S. iranensis* ∆*SIRAN5116* | *SIRAN5116*::*aac(3)IV*, *oriT* | This study |
| *S. iranensis* ∆*razA* | *SIRAN1138*::*aac(3)IV*, *oriT* | This study |
| *S. iranensis* ∆*razB* | *SIRAN1137*::*aac(3)IV*, *oriT* | This study |
| *S. iranensis* ∆*razD* | *SIRAN1135*::*aac(3)IV*, *oriT* | This study |
| *S. iranensis* ∆*razA*^c^ | *SIRAN1138*::*aac(3)IV*, *oriT;* complemented with *pIJ razA* | This study |
| *S. iranensis* ∆*razB*^c^ | *SIRAN1137*::*aac(3)IV*, *oriT*; complemented with *pIJ* np_*razB* | This study |
| *S. iranensis* ∆*razD*^c^ | *SIRAN1135*::*aac(3)IV*, *oriT*; complemented with *pIJ razD* | This study |
| *S. iranensis* ∆*razA* pIJ *razD* | *SIRAN1138*::*aac(3)IV*, *oriT*; complemented with *pIJ razD* | This study |
| *S. iranensis* ∆*razB* pIJ *razD* | *SIRAN1137*::*aac(3)IV*, *oriT*; complemented with *pIJ razD* | This study |
| *S. iranensis* ∆*razA* pIJ10257 | *SIRAN1138*::*aac(3)IV*, *oriT*; with empty vector as control | This study |
| *S. iranensis* ∆*razB* pIJ10257 | *SIRAN1137*::*aac(3)IV*, *oriT*;  with empty vector as control | This study |
| *E. coli* DH10β | Δ(ara-leu) 7697, araD139, fhuA, ΔlacX74, galK16, galE15, e14^-^, ϕ80dlacZΔM15, recA1, relA1, endA1, nupG, rpsL (StrR), rph spoT1, Δ(mrr-hsdRMS-mcrBC) | New England Biolabs, Frankfurt, Germany |
| *E. coli* BW25113 pIJ790 | acIq, rrnBT14, ∆lacZWJ16, hsdR514, ∆araBA-, DAH33, ∆rhaBADLD78 | Datsenko and Wanner, 2000 |
| *E. coli* ET12567 pUZ8002 | Fˉ, dam-13::Tn9, dcm-6, hsdR, zij- 202 ::Tn10, recF143, galK2, galT22, ara-14, lacY1, xyl-5, leuB6, thi-1, tonA31, rpsL136, HisG4, tsx-78, mtl- 1, glnV44 | MacNeil et al., 1992 |
| *E. coli* BL21(DE3) | F^–^, ompT, hsdS_B_, (r_B_–, m_B_–), gal, dcm (DE3) | New England Biolabs,  Frankfurt, Germany |

**TABLE S4** Plasmids used in this study.

| Plasmid | Relevant features | Reference |
| --- | --- | --- |
| pIJ10257 | Hygromycin resistance gene; *ermE** promoter, ΦBT1 phage integration site, *oriT* | Hong *et al*., 2005 |
| pIJ *razA* | pIJ10257 with *razA* under control of the *ermE** promoter | This study |
| pIJ np_*razB* | pIJ10257 with *razB* and its putative promoter region | This study |
| pIJ *razD* | pIJ10257 with *razD* under control of the *ermE** promoter | This study |
| pKOSi | *kanR,* pSG5 replicon, *oriT* | Netzker *et al*., 2016 |
| pKOSi *∆SIRAN5116* | pKOSi with upstream and downstream regions of *SIRAN5116* flanking aac(3)IV | This study |
| pKOSi ∆*razA* | pKOSi with upstream and downstream regions of *razA* flanking aac(3)IV | This study |
| pKOSi ∆*razB* | pKOSi with upstream and downstream regions of *razB* flanking aac(3)IV | This study |
| pKOSi ∆*razD* | pKOSi with upstream and downstream regions of *razD* flanking aac(3)IV | This study |
| pET28a | Expression of target proteins | Novagen Merck, Darmstadt, Germany |

**TABLE S5** Antibiotics used in this study and working concentrations.

| Antibiotic | Working concentration |
| --- | --- |
| apramycin | 50 µg/ml |
| hygromycin | 50 µg/ml |
| kanamycin | 25 µg/ml for *E. coli* 50 µg/ml |
| Nalidixic acid | 25 µg/ml |

**TABLE S6** Oligonucleotides used in this study.

| Oligo-nucleo-tide | Sequence (5’-3’), restriction sites underlined | Restriction enzymes and the purpose of oligonucleotides |
| --- | --- | --- |
| P1 | CTCGAATCTAGAGCCTGATCGTCTCCATAGTCG | *Bgl*II, cloning of the knock out plasmid pKOSi ∆*SIRAN5116* |
| P2 | GCGAAACACCGACACTAGGC | Cloning of the knock out plasmid pKOSi ∆*SIRAN5116* |
| P3 | CATGCGTGCGGGGCGCGACGAGGCGAGGGGAACCGCATGATTCCGGGGATCCGTC GACC | Cloning of the knock out plasmid pKOSi ∆*SIRAN5116* |
| P4 | CATCTGCCGCACCCTTGGTCCCGCGAAACCACTTACTCATGTAGGCTGGAGCTGCTT C | Cloning of the knock out plasmid pKOSi ∆*SIRAN5116* |
| P5 | CTCGAATCTAGAACAGCTCATAGCTCCAGTCG | *Bgl*II, cloning of the knock out plasmid pKOSi ∆*razA* |
| P6 | CGTTCAAGCAACATCGATTCCG | Cloning of the knock out plasmid pKOSi ∆*razA* |
| P7 | CCGAAATTCTTCGCATTTTGTGGCTTGGGAGGCTGCGTGATTCCGGGGATCCGTCGACC | Cloning of the knock out plasmid pKOSi ∆*razA* |
| P8 | GGGTCGTGAACCTGTCAGCGAACCGCACTGCGCGCCTCATGTAGGCTGGAGCTGCTTC | Cloning of the knock out plasmid pKOSi ∆*razA* |
| P9 | CTCGTTAAGCTTCGTCATTACGCTCGTGTAGC | *Hin*dIII, cloning of the knock out plasmid pKOSi ∆*razB* |
| P10 | CGCAATAGATCCGTGTACTCG | Cloning of the knock out plasmid pKOSi ∆*razB* |
| P11 | CAGGTTCACGACCCATCCCAGGCATATAGTCACACCAT GATTCCGGGGATCCGTCGA CC | Cloning of the knock out plasmid pKOSi ∆*razB* |
| P12 | CGCGACCTGCGCCAGCTCGGCCTCCCGTTCAAGCAAC | Cloning of the knock out plasmid pKOSi ∆*razB* |
| P13 | GCTATGGACGATCTGTCAACGA | Cloning of the knock out plasmid pKOSi ∆*razD* |
| P14 | CTCGAATCTAGACTCGATGACCGTGGCCGAAC | *Bgl*II, cloning of the knock out plasmid pKOSi ∆*razD* |
| P15 | TCCGAATACTGAGAATTAGGAAGGGATGGCAGTCCCTTGATTCCGGGGATCCGTCGACC | Cloning of the knock out plasmid pKOSi ∆*razD* |
| P16 | GCCGTGGCGCGTCCGTGGACCTCCGCGACGCGGAGGTCATGTAGGCTGGAGCTGCTTC | Cloning of the knock out plasmid pKOSi ∆*razD* |
| P17 | TTACATATGGACCGGAAAACGGATTCCGGT | *Nde*I, cloning of the complementation plasmid pIJ *razA* |
| P18 | CTTAAGCTTTCAGCGTCGGCCGCGTGA | *Hin*dIII, cloning of the complementation plasmid pIJ *razA* |
| P19 | TCTGGTACCGGCGCGCAGTGCGGTTCGCT | *Kpn*I, cloning of the complementation plasmid pIJ np_*razB* |
| P20 | ATTAAGCTTTCAAGCAACATCGATTCCGCCTC | *Hin*dIII, cloning of the complementation plasmid pIJ np_*razB* |
| P21 | TTTCATATGGCATTGCCTTTACTAGCGGA | *Nde*I, cloning of the complementation plasmid pIJ *razD* |
| P22 | TTGAAGCTTCATTTGACGAAGTCGTCGAC | *Hin*dIII, cloning of the complementation plasmid pIJ *razD* and pIJ np_*razD* |
| P23 | CCTTCACATACTTGTCGTCCAGC | EMSA-Primer for negative control |
| P24 | GCAAGGAGCTCTACCTCAAGG | 5’-Cyanine 3 labelled EMSA-Primer for negative control |
| P25 | GGAAATCGGCAGGCCATACCG | 5’-Cyanine 3 labelled EMSA-Primer for fragment A |
| P26 | CCTGGCGCGTGAAAACAGC | EMSA-Primer for fragment A |
| P27 | GGAAATCGGCAGGCCATACCG | 5’-Cyanine 3 labelled EMSA-Primer for fragment A.1 |
| P28 | CGTTAAGTGTGGAGTTTCTGATGC | EMSA-Primer for fragment A.1 |
| P29 | GCATCAGAAACTCCACACTTAACG | 5’-Cyanine 3.5 labelled EMSA-Primer for fragment A.2 |
| P30 | GCTGATGAAACGCTACTGAAACC | EMSA-Primer for fragment A.2 |
| P31 | GGTTTCAGTAGCGTTTCATCAGC | 5’-Cyanine 3 labelled EMSA-Primer for fragment A.3 |
| P32 | CCTGGCGCGTGAAAACAGC | EMSA-Primer for fragment A.3 |
| P33 | GCATCAGAAACTCCACACTTAACG | 5’-Cyanine 3 labelled EMSA-Primer for fragment A.2.1 |
| P34 | CTTATGGGACCGCGGCTAGTG | EMSA-Primer for fragment A.2.1 |
| P35 | CACTAGCCGCGGTCCCATAAG | 5’-Cyanine 3 labelled EMSA-Primer for fragment A.2.2/positive control |
| P36 | GCTGATGAAACGCTACTGAAACC | EMSA-Primer for fragment A.2.2/positive control |
| P37 | CACTAGCCGCGGTCCCATAAGGGAAACCCT | 5’-Cyanine 3 labelled EMSA-Primer for fragment A.2.2.1 |
| P38 | AGGGTTTCCCTTATGGGACCGCGGCTAGTG | EMSA-Primer for fragment A.2.2.1 |
| P39 | GGGAAACCCTAGAAACCCACCCAGGCATGG | 5’-Cyanine 3 labelled EMSA-Primer for fragment A.2.2.2 |
| P40 | CCATGCCTGGGTGGGTTTCTAGGGTTTCCC | EMSA-Primer for fragment A.2.2.2 |
| P41 | CCAGGCATGGCTGACAACCCCGGGCAGCGT | 5’-Cyanine 3 labelled EMSA-Primer for fragment A.2.2.3 |
| P42 | ACGCTGCCCGGGGTTGTCAGCCATGCCTGG | EMSA-Primer for fragment A.2.2.3 |
| P43 | CGGGCAGCGTTCCGGGGATTTGGTTTCAGT | 5’-Cyanine 3 labelled EMSA-Primer for fragment A.2.2.4 |
| P44 | ACTGAAACCAAATCCCCGGAACGCTGCCCG | EMSA-Primer for fragment A.2.2.4 |
| P45 | GGGATTTGGTTTCAGTAGCGTTTCATCAGC | 5’-Cyanine 3 labelled EMSA-Primer for fragment A.2.2.5 |
| P46 | GCTGATGAAACGCTACTGAAACCAAATCCC | EMSA-Primer for fragment A.2.2.5 |
| P47 | CGCGGTCCCATAAGGGAAACCC | 5’-Cyanine 3 labelled EMSA-Primer for fragment A.2.2.6 |
| P48 | GGGTTTCCCTTATGGGACCGCG | EMSA-Primer for fragment A.2.2.6 |
| P49 | GGTCCCATAAGGGAAACCCTAGAAACCCAC | 5’-Cyanine 3 labelled EMSA-Primer for fragment A.2.2.7 |
| P50 | GTGGGTTTCTAGGGTTTCCCTTATGGGACC | EMSA-Primer for fragment A.2.2.7 |
| P51 | AGAAACCCACCCAGGCATGGCTGACAACCC | 5’-Cyanine 3 labelled EMSA-Primer for fragment A.2.2.8 |
| P52 | GGGTTGTCAGCCATGCCTGGGTGGGTTTCT | EMSA-Primer for fragment A.2.2.8 |
| P53 | TGAGCGGGGAGCGGCCGA | 5’-Cyanine 3 labelled EMSA-Primer for upstream region of *SIRAN_RS26945* |
| P54 | GGTGACTCCCGAAGTCCTCGCGA | EMSA-Primer for upstream region of *SIRAN_RS26945* |
| P55 | AAAACACCGGCAGAAAACCTAGTGTCCTGG | 5’-Cyanine 3 labelled EMSA-Primer for upstream region of *SIRAN_RS02585* |
| P56 | CCAGGACACTAGGTTTTCTGCCGGTGTTTT | EMSA-Primer for upstream region of *SIRAN_RS02585* |
| P57 | GATGATGTCCTGCATGACTTGTCC | 5’-Cyanine 3 labelled EMSA-Primer for upstream region of *SIRAN_RS06010* |
| P58 | GCACGCTCATCGCCTGTCTG | EMSA-Primer for upstream region of *SIRAN_RS06010* |
| P59 | AAGACCAGGGAGCCTAGGGGGGA | 5’-Cyanine 3 labelled EMSA-Primer for upstream region of *SIRAN_RS30355* |
| P60 | CGAGTCGAGCGCGTAGATCTTCG | EMSA-Primer for upstream region of *SIRAN_RS30355* |
| P61 | CTCGTCACGGGAGAAACTGTTCG | 5’-Cyanine 3 labelled EMSA-Primer for upstream region of *SIRAN_RS30365* |
| P62 | GGTTGACCACGCGCACTTCCTCC | EMSA-Primer for upstream region of *SIRAN_RS30365* |
| P63 | CGCTTCATCATCTCCTCTCTTCGTCACA | 5’-Cyanine 3 labelled EMSA-Primer for upstream region of *SIRAN_RS22710* |
| P64 | TTCGAGGTCGAGGCCGTCCAAC | EMSA-Primer for upstream region of *SIRAN_RS22710* |
| P65 | TCCAGGAGGTCTAGCGGGGAGT | 5’-Cyanine 3 labelled EMSA-Primer for upstream region of *SIRAN_RS13165* |
| P66 | GAGAACCGTCCTATTCGCCCGAA | EMSA-Primer for upstream region of *SIRAN_RS13165* |
| P67 | TGGTTGCTACGCATTTCCCTAGCGATTGCC | 5’-Cyanine 3 labelled EMSA-Primer for upstream region of *SIRAN_RS03185* |
| P68 | GGCAATCGCTAGGGAAATGCGTAGCAACCA | EMSA-Primer for upstream region of *SIRAN_RS03185* |
| P69 | GGTTGCTCCGGAAATCCCCTAGCTTCCCGC | 5’-Cyanine 3 labelled EMSA-Primer for upstream region of *SIRAN_RS18590* |
| P70 | GCGGGAAGCTAGGGGATTTCCGGAGCAACC | EMSA-Primer for upstream region of *SIRAN_RS18590* |
| P71 | CCGAAAACCCGGAATTCCCTAGGAATAGAA | 5’-Cyanine 3 labelled EMSA-Primer for upstream region of *SIRAN_RS08520* |
| P72 | TTCTATTCCTAGGGAATTCCGGGTTTTCGG | EMSA-Primer for upstream region of *SIRAN_RS08520* |
| P73 | CGTCAGCCGCTGCCTCCAGC | 5’-Cyanine 3 labelled EMSA-Primer for upstream region of *SIRAN_RS20480* |
| P74 | ACTCACACGCTCTTTCGTCG | EMSA-Primer for upstream region of *SIRAN_RS20480* |
| P75 | GCAATGCCAAGGGACTGCCA | 5’-Cyanine 3.5 labelled EMSA-Primer for upstream region of *razD* |
| P76 | GCATTCGTGAACGGAACGTT | EMSA-Primer for upstream region of *razD* |

**References**

Datsenko, K., and Wanner, B. (2000) One-step inactivation of chromosomal genes in *Escherichia coli* K-12 using PCR products. *Proc. Natl. Acad. Sci*. **97**, 6640–6645.

Hamedi, J., Mohammadipanah, F., Klenk, H.-P., Pötter, G., Schumann, P., Spröer, C., and von Jan, M., Kroppenstedt, R. (2010) *Streptomyces iranensis* sp. nov., isolated from soil. *Int. J. Syst. Evol. Microbiol.* **60**, 1504–1509.

Hong, H.-J., Hutchings, M., Hill, L., and Buttner, M. (2005) The role of the novel fem protein VanK in vancomycin resistance in *Streptomyces coelicolor*. *J. Biol. Chem*. **280**, 13055–13061.

MacNeil, D., Gewain, K., Ruby, C., Dezeny, G., Gibbons, P., and MacNeil, T. (1992) Analysis of *Streptomyces avermitilis* genes required for avermectin biosynthesis utilizing a novel integration vector. *Gene* **111**, 61–68.

Netzker, T., Schroeckh, V., Gregory, M., Flak, M., Krespach, M., Leadlay, P., and Brakhage, A. A. (2016) An efficient method to generate gene deletion mutants of the rapamycin-producing bacterium *Streptomyces iranensis* HM 35. *Appl. Environ. Microbiol*. **82**, 3481–3492.

Santos-Aberturas, J., Payero, T., Vicente, C., Guerra, S., Cañibano, C., Martín, J., Aparicio, J., 2011. Functional conservation of PAS–LuxR transcriptional regulators in polyene macrolide biosynthesis. *Metab. Eng*. **13**, 756–767.
